# Multivalent viral particles elicit safe and efficient immunoprotection against Nipah Hendra and Ebola viruses

**DOI:** 10.1038/s41541-022-00588-5

**Published:** 2022-12-17

**Authors:** Duncan G. Ithinji, David W. Buchholz, Shahrzad Ezzatpour, I. Abrrey Monreal, Yu Cong, Julie Sahler, Amandip Singh Bangar, Brian Imbiakha, Viraj Upadhye, Janie Liang, Andrew Ma, Birgit Bradel-Tretheway, Benjamin Kaza, Yao Yu Yeo, Eun Jin Choi, Gunner P. Johnston, Louis Huzella, Erin Kollins, Saurabh Dixit, Shuiqing Yu, Elena Postnikova, Victoria Ortega, Avery August, Michael R. Holbrook, Hector C. Aguilar

**Affiliations:** 1 School for Global Animal Health, Washington State University, Pullman, WA USA; 2 Kenya Agricultural and Livestock Research Organization, Nairobi, Kenya; 3 Department of Microbiology and Immunology, Cornell University, Ithaca, NY USA; 4 National Institute of Allergy and Infectious Diseases (NIAID) Integrated Research Facility, Ft Detrick, Frederick, MD 21702 USA

**Keywords:** Protein vaccines, Viral infection

## Abstract

Experimental vaccines for the deadly zoonotic Nipah (NiV), Hendra (HeV), and Ebola (EBOV) viruses have focused on targeting individual viruses, although their geographical and bat reservoir host overlaps warrant creation of multivalent vaccines. Here we explored whether replication-incompetent pseudotyped vesicular stomatitis virus (VSV) virions or NiV-based virus-like particles (VLPs) were suitable multivalent vaccine platforms by co-incorporating multiple surface glycoproteins from NiV, HeV, and EBOV onto these virions. We then enhanced the vaccines’ thermotolerance using carbohydrates to enhance applicability in global regions that lack cold-chain infrastructure. Excitingly, in a Syrian hamster model of disease, the VSV multivalent vaccine elicited safe, strong, and protective neutralizing antibody responses against challenge with NiV, HeV, or EBOV. Our study provides proof-of-principle evidence that replication-incompetent multivalent viral particle vaccines are sufficient to provide protection against multiple zoonotic deadly viruses with high pandemic potential.

## Introduction

The current COVID-19 pandemic has had huge devastating impact on global health and economy^1^. The mechanisms of COVID-19 transmission are shared among many other viruses^2,3^. This pandemic emphasizes the need for the global community to be prepared for virus disease outbreaks, such as by the preemptive development of prophylactic vaccines. Some of the deadliest viruses of concern are the subject of this study.

Nipah virus (NiV), Hendra virus (HeV), and Ebola virus (EBOV) are highly lethal zoonotic viruses requiring biosafety level 4 (BSL-4) containment. NiV and HeV cause encephalitis and respiratory disease in humans and susceptible animals^4–6^ with a mortality rate of 40–100%^5,7,8^. EBOV causes a multisystem disease in humans and nonhuman primates^9^, with a mortality rate up to 90% in humans. Diseases caused by NiV, HeV, and EBOV have huge impacts on human and animal health. The first Asian NiV outbreak in 1998–1999 alone led to the slaughter of more than a million pigs and >100 human deaths^10^. The 2014 EBOV outbreak in West Africa caused >11,000 human deaths from >28,000 cases^11^ which led the World Health Organization to declare the outbreak a Public Health Emergency of International Concern (PHEIC)^11,12^. Nipah virus has caused multiple outbreaks with high mortality rates (averaging ~75%) in Asia^10,13–17^, as has HeV (averaging ~40%) in Australia^4^ and EBOV (averaging ~50%) in Africa^9,13^.

**Fig. 1:**
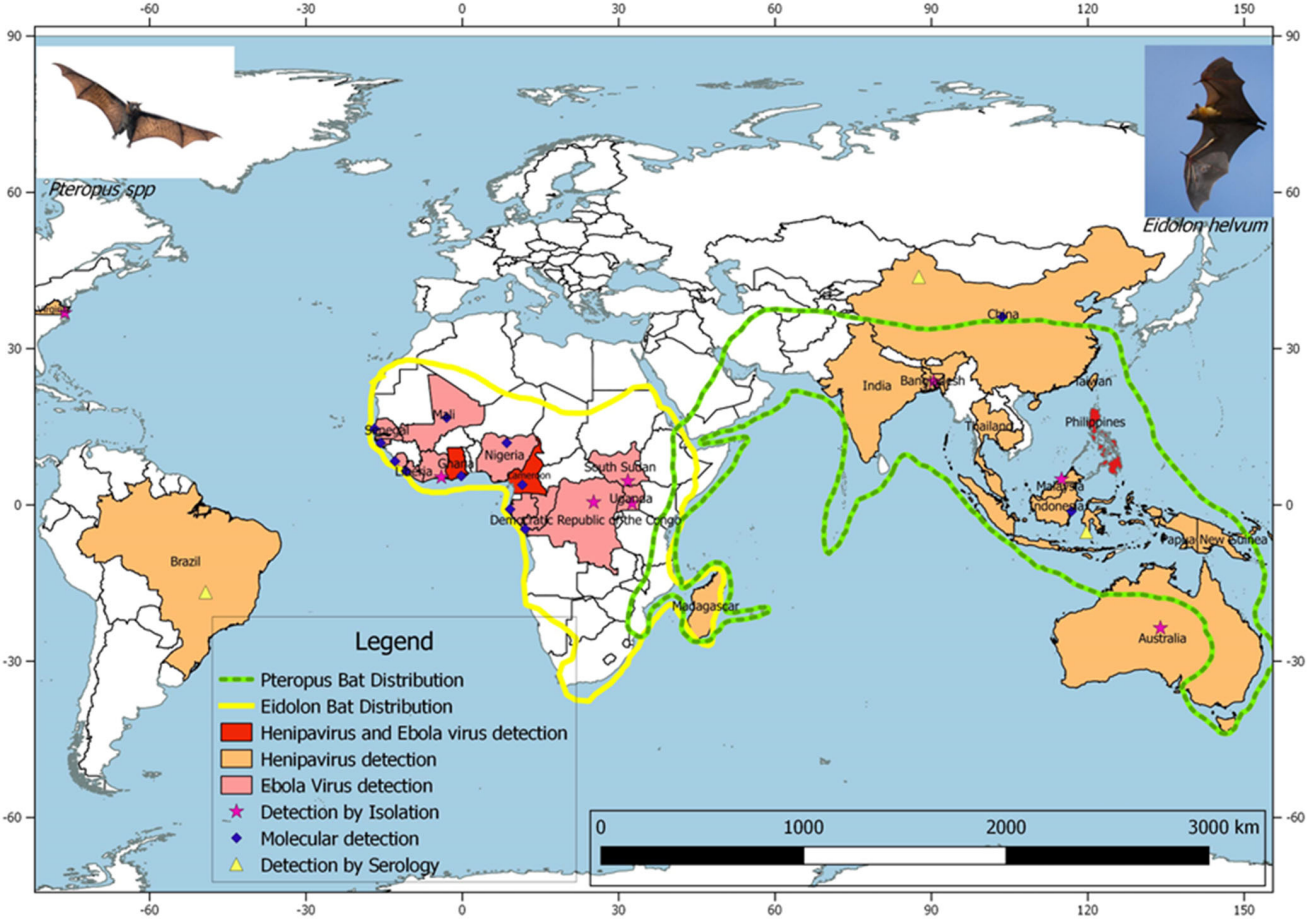
Map showing the detection of Henipavirus and Ebola virus, and the distribution of their most likely reservoir host—the Pteropus and Eidolon bat genera. The green dotted line shows the distribution of Pteropus bats while the continuous yellow line shows distribution of the Eidolon bat species. Determination of the virus distribution is by detection of Viral RNA or antibodies against the viruses. The orange shade indicates countries where Henipavirus outbreaks have been recorded or henipaviruses detected in bats, while the pink shade indicates outbreaks or detection of Ebola virus. The red shade shows detection and/or outbreaks for both Ebola and henipaviruses.

Much evidence supports the notion that the main reservoir host for all three viruses are fruit bats in the family *Pteropodidae*, such as those in the genera *Pteropus* and *Eidolon*. However, evidently henipaviruses and EBOV are spreading into newer territories. This is associated with the spreading distribution of fruit bats caused by deforestation, climate change, and human movement^18,19^ (Fig. 1). A number of countries have reported henipavirus outbreaks or are at risk based on serological or molecular detection in *Pteropus* bats^20^ and the home range of *Pteropus* bats being widely spread (CDC 2014). For instance, antibodies against henipaviruses have been detected in Ghanaian bats and Cameroon human samples in West Africa^18,19^. The distribution of these reservoir hosts helps predict the potential origin of these viral diseases (Fig. 1) and calls for a common vaccine platform for combating them, providing the motive for this study.

Henipavirus genomes consist of six genes N, P, M, F, G, and L, which encode nine proteins^21,22^. The receptor binding glycoprotein (G) and fusion glycoprotein (F) facilitate viral attachment and entry into cells, respectively^23^, and are key antigens in generating neutralizing antibody responses^24^. Ebolaviruses encode nine proteins NP, GP, soluble GP (sGP), small soluble GP (ssGP), L and four structural proteins termed VP24, VP30, VP35, and VP40^25^. The EBOV glycoprotein (GP) is the only virally expressed protein on the virion surface and is critical for attachment to host cells and execution of membrane fusion during viral entry^26^. The EBOV GP should be a vital component of vaccines, as it is targeted by neutralizing antibodies and inhibitors of attachment and fusion. GP, sGP, and ssGP are produced from the GP gene by alternative RNA editing^25^.

Although there are currently no NiV and HeV vaccines licensed for human use, a recombinant replication-competent VSV vaccine has been produced and licensed in the United States for Ebola virus^11,27,28^ and a soluble G glycoprotein vaccine for HeV is available for animal use in Australia^5^. Monovalent Nipah virus-like particles (VLPs) have been produced, incorporating attachment and fusion glycoproteins^29^. VLPs are safe as they are replication incompetent, present the viral glycoproteins in their native conformation and are generally good immunogens, and their use also allows differentiating infected from vaccinated animals (DIVA) which is critical in areas where animal vaccination may be implemented^30^. The VSV platform has been used for production of several vaccines^31^ which are either replication competent or incompetent.

In this study, we sought to determine whether two or more surface glycoproteins from different viruses can be incorporated onto VLPs or VSV pseudotyped virions for use as candidate multivalent VLP-based vaccines. We produced and characterized multivalent NiV-HeV-EBOV VLPs and pseudotyped VSV virions, tested using a Syrian hamster model of Henipavirus and Ebolavirus disease^32^. We also analyzed the thermostability of the VLP and pseudotyped VSV vaccines and lyophilized them in the presence of carbohydrates to improve their thermotolerance^33^. Lyophilization will improve field vaccine administration, as these viruses cause disease in regions of the world that face challenges for maintaining vaccine cold-chain. The replication-incompetent VSV multivalent vaccine showed superior incorporation of the glycoproteins and 100% efficacy and safety upon challenge with any of the three NiV, HeV, or EBOV.

## Results

### Multivalent VLPs are effectively produced using HEK 293T cells

**Fig. 2:**
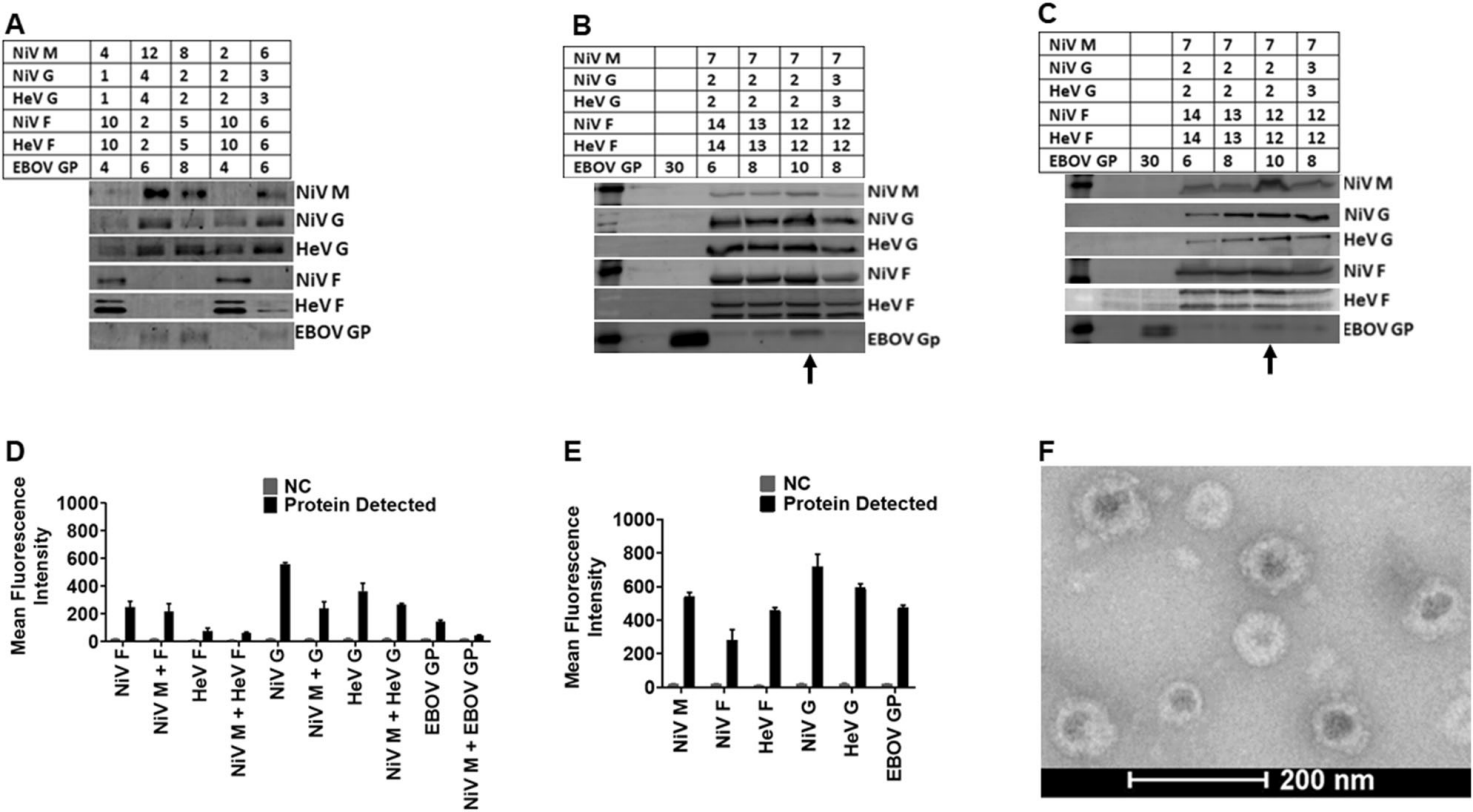
Optimized production of NiV-HeV-EBOV VLPs and their protein detection using multiple techniques. **A** Different amounts of DNA (µg) were used to transfect HEK 293T cells to determine the closest range for each target protein for optimized production detected by Western Blot analysis (WBA). **B** Shows the best VLP production DNA ratio detected by WBA. **C** WBA detection of proteins in cell lysate of cells transfected to produce VLPs in (**B**). **D** The cell surface expression (CSE) of the target proteins analyzed by Flow Cytometry (FC). **E** Detection of the target proteins on VLPs using Flow Virometry (FV). **F** A micrograph of the produced VLPs viewed using a FEI T20 electron microscope.

To determine the optimal incorporation of proteins onto VLPs, we transfected human embryonic kidney cells (HEK 293T cells) with different ratios of expression plasmid DNA for NiV M, F, G (Malaysia strain), HeV F, G (original Australian strain), and EBOV GP (Zaire strain), for a total of 30 µg of plasmid DNA per 15-cm dish (Fig. 2A). Incorporation of proteins onto the VLPs was confirmed using standard Western blot analysis. From the initial results of experiments such as that shown in Fig. 2A, we expanded the total µg of DNA used to 45 µg/15-cm dish and tried various DNA ratios (Fig. 2B). Transfection of cells with the DNA ratio 7:12:12:2:2:10 µg for NiV M, NiV F, HeV F, NiV G, HeV G, and EBOV GP, respectively, yielded the best protein incorporation into virions (Fig. 2B) and expression of the proteins in cell lysates (Fig. 2C), determined by fluorescent Western blot analysis quantification. Individual protein blots with molecular weight markers for VLPs are shown in supplementary Fig. 11. We also determined the cell surface expression (CSE) levels for each glycoprotein by flow cytometry (Fig. 2D). Incorporation of the proteins onto VLPs was determined by flow virometry^34^ (Fig. 2E). The results demonstrated that multiple glycoproteins can be found on the surfaces of viral particles of a single viral preparation. We demonstrated the presence and distribution of the glycoproteins on the surfaces of VLPs by electron microscopy. Figure 2F shows a TEM micrograph of VLPs and their spikes, which were not observed in negative control bald particle samples. These figures demonstrate that different immunogenic glycoproteins can be incorporated onto VLPs following transfection of HEK 293T cells.

### Multivalent pseudotyped VSV particles are effectively produced using HEK 293T cells

**Fig. 3:**
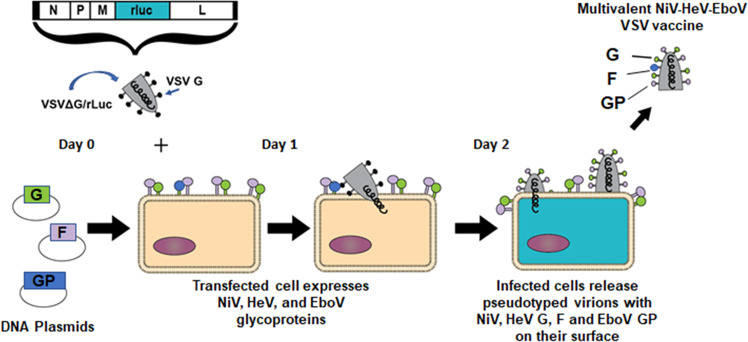
Pseudotyped virion production system. A diagram showing transfection of HEK 293T cells using plasmids carrying DNA encoding for the immunogenic proteins of interest, followed by infection using VSV-deltaG virus. The subsequent pseudotyped VSV virion preparation carries all five distinct viral glycoproteins and can be used as a multivalent vaccine.

Similarly, as in Fig. 2, we transfected HEK 293T cells with varying amounts of expression plasmid DNA for NiV F/G, HeV F/G, and EBOV GP for 12 h and then infected them with VSV-rLuc virions as was applied in previous studies and depicted in Fig. 3^35,36^.

**Fig. 4:**
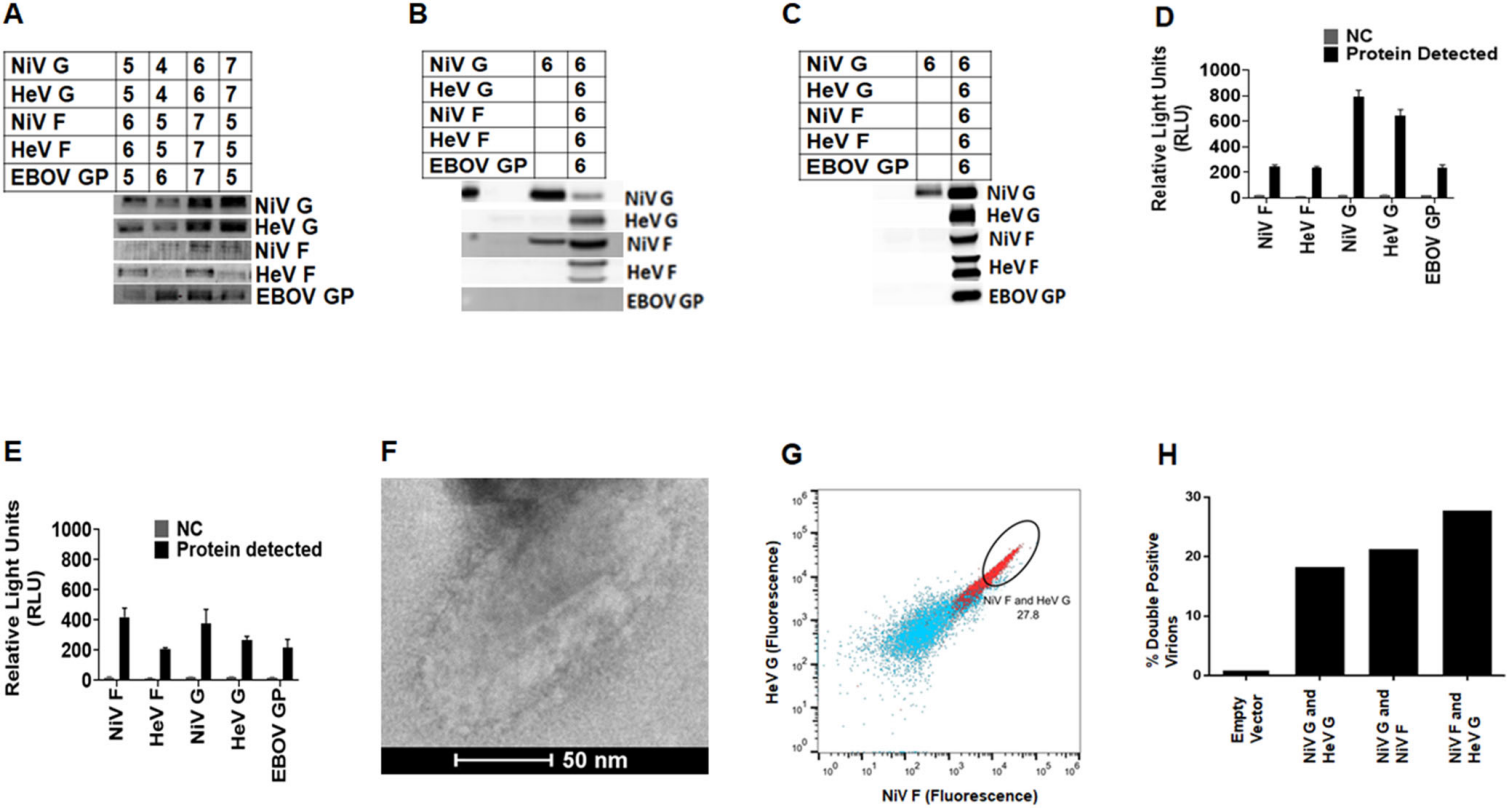
Optimized production of Pseudotyped VSV incorporating NiV-HeV-EBOV glycoproteins. **A** Different amounts of DNA (µg) were used to give the closest range for each target protein for optimized production detected by WBA. **B** WBA detection of proteins in cell lysate of cells transfected to produce the pseudotyped VSV particles. **C** Shows the best DNA ratio for production of pseudotyped VSV detected by WBA. **D** The cell surface expression (CSE) of the target proteins analyzed by FC. **E** Detection of the target proteins on pseudotyped VSV using FV. **F** A micrograph of the produced pseudotyped VSV particles viewed using a FEI T20 electron microscope. **G** Flow cytometry data showing an example of the presence of double-positive viral populations (red) as compared to the negative control bald particles (blue). **H** Percentage of viral populations double positive for various pairs of glycoproteins with extracellular tags. One representative experiment of three is shown.

From the initial Western blot analysis (Fig. 4A), we decided that the best pseudotyped VSV protein incorporation would be achieved after transfection with DNA amounts of about 5 to 7 µg for each protein expression plasmid. We therefore transfected the HEK 293T cells with 6 µg DNA to express each of the NiV, HeV, and EBOV surface glycoproteins (Fig. 4B) for incorporation onto the pseudotyped VSV. The proteins were also detected in the cell lysates by Western blot analysis (Fig. 4C). The Western blots with molecular markers for the detection of Pseudotyped VSV particles and lysates are shown in supplementary Fig. 11. The cells used to produce pseudotyped VSV particles were stained in a similar manner to those used for VLP production. The levels of glycoprotein incorporation were determined by flow virometry (Fig. 4D), and expression of the glycoproteins on the pseudotyped VSV was also determined by flow cytometry (Fig. 4E). Multiple glycoproteins were found on individual viral particles. Figure 4F shows a TEM micrograph of a pseudotyped VSV particle showing surface glycoproteins, again, not observed in negative control samples. We also demonstrated the presence of more than one type of glycoprotein on the surface of individual virus particles by flow virometry (Fig. 4G, H). Figure 4G shows an example of the gating of individual viral particles containing both NiV F and HeV G on single particles. The same gating strategy was used to determine the percentage of double-positive virions for various other pairs of glycoproteins that contained extracellular (extraviral) tags (Fig. 4H).

### Trehalose lyophilization improves viral particle thermostability

**Fig. 5:**
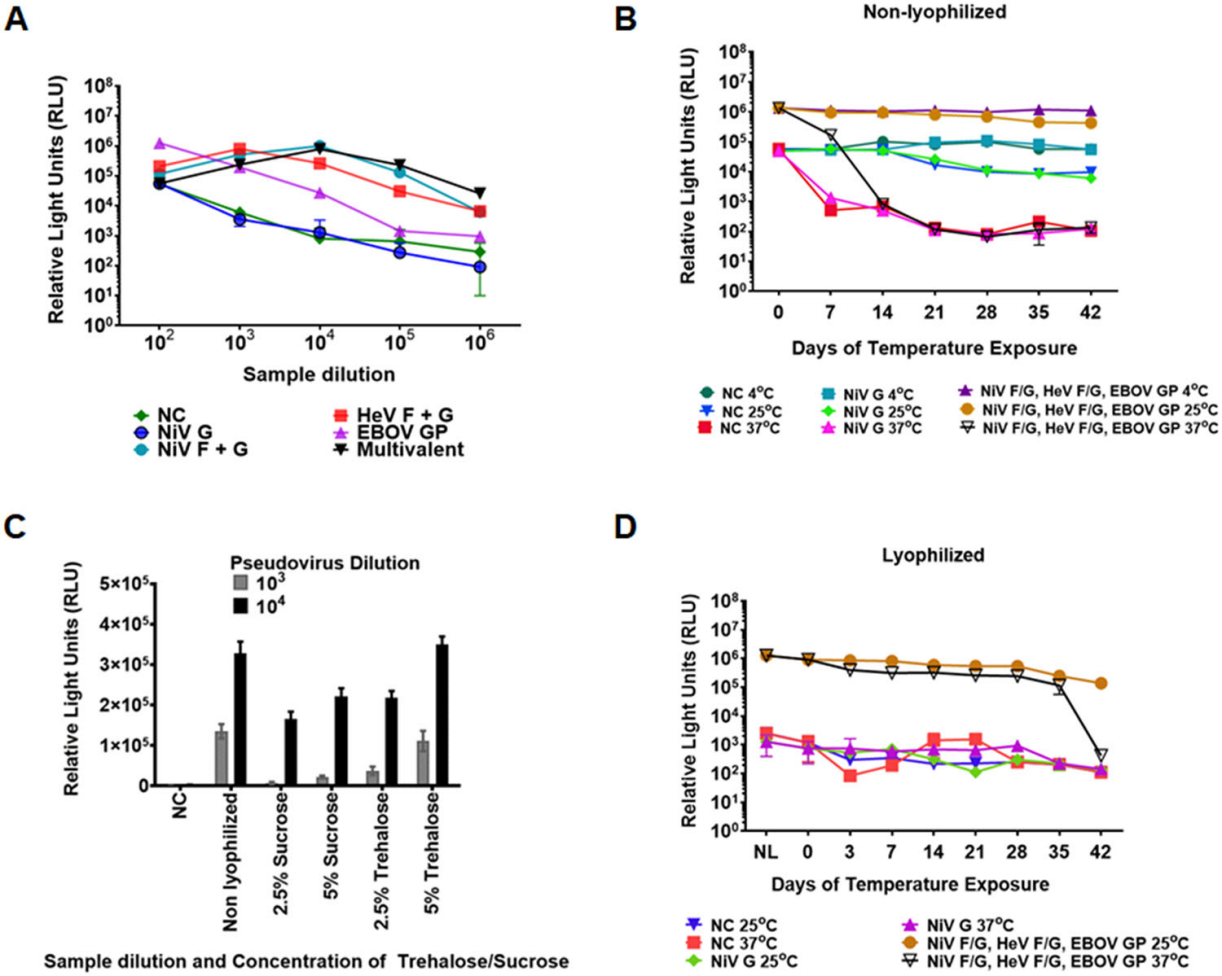
Carbohydrates preserve the thermostability of pseudotyped VSV particles. **A** Vero cell entry capabilities for pseudotyped VSV incorporating monovalent and multivalent target proteins determined using Renilla Luciferase assay. The relative MOI of the pseudotyped VSV was determined based on luminescence produced from the Luc gene and read as relative light units (RLU). The pseudotyped VSV virions were collected 40 h post transfection. The monovalent and multivalent pseudotyped VSV virions were diluted 1:100 to 1:1,000,000 and their entry capabilities determined to assess the optimal entry levels for the monovalent and multivalent pseudotyped VSV. **B** Determination of Vero cell entry of non-lyophilized multivalent pseudotyped VSV exposed to 4 °C, 25 °C, and 37 °C for 42 days. **C** Determination of sucrose and trehalose capability to preserve the multivalent pseudotyped VSV during lyophilization. **D** Determination of viability for the 5% w/v trehalose preserved pseudotyped VSV. Viability was determined using the Renilla Luciferase assay.

To determine the thermostability of the pseudotyped virions, we used the cell entry capabilities of the monovalent and multivalent VSV virions as a surrogate of particle stability. Their luciferase marker gene allowed us to assess viral particle entry. We made pseudotyped VSV virions incorporating NiV G only (negative control for viral entry), NiV F/G, HeV F/G, EBOV GP only, or all NiV F/G, HeV F/G and EBOV GP (multivalent virions). Bald virions with plasmids pCDNA3.1/pCAGGS served as an additional negative control for viral entry. The pseudotyped virions were used to infect Vero cells at a confluency of 30% and VSV luminescence was recorded 24 h post-infection (hpi). Figure 5A shows the cell entry levels of the monovalent and the multivalent virions.

After determining the entry capabilities of the pseudotyped virions, we then determined their stability under different temperature environments. We exposed the multivalent virions to temperatures of 4 °C, 25 °C, and 37 °C for a period of up to six weeks and determined their entry levels into Vero cells, as in Fig. 5A. The virions were stable at 4 °C and 25 °C for the six weeks but lost a log in luminescence detection within a week when exposed to 37 °C, and luminescence detection was in the range of the negative controls by the end of the second week (Fig. 5B).

Thermotolerance of virus vaccines has been enhanced using carbohydrates^37^. We compared the capacity of sucrose and trehalose to preserve the multivalent pseudotyped virions following a lyophilization process. The virions were lyophilized in 2.5% or 5% sucrose or trehalose for 24 h at 1:100 and 1:1000 dilutions. Following lyophilization, 5% trehalose preserved the pseudotyped virions completely, yielding luciferase levels similar to those of fresh non-lyophilized virions (Fig. 5C). We then lyophilized the multivalent pseudotyped virions using 5% trehalose and exposed them to temperatures of 4 °C, 25 °C, and 37 °C. Positive control lyophilized virions were preserved under vacuum at 4 °C for the six-week period, while we placed vials of virions under three different temperatures starting with the day 42 vials, and ending with the day 0 vials (we placed the day 42 vials first, then day 35 vials after a week, then days 28, 21, 14, and 7 in that order), so that all vials were tested on the same day (day 0). The Day 0 lyophilized sample (Fig. 5D) represents values for vials at kept at 4 °C for the entire period. There was minimal loss in viability of the pseudotyped virions following lyophilization when stored at 4 °C or 25 °C, as measured by viral entry levels. Remarkably, we observed a minimal loss in luminescence for the lyophilized virions for up to five weeks at 37 °C, a high environmental temperature. Therefore, lyophilization in 5% trehalose improved thermotolerance of the pseudotyped VSV virions, allowing the use of this type of vaccine in high-temperature climates where maintenance of a cold-chain is impractical.

### The multivalent VLPs elicited neutralizing antibody responses in hamsters

**Fig. 6:**
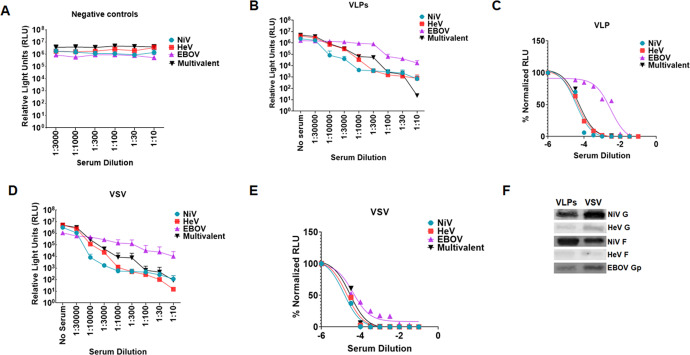
Multivalent VLPs and pseudotyped VSV incorporating NiV F/G, HeV F/G, and EBOV GP elicited neutralizing antibodies in hamsters. **A**. Negative control hamsters were vaccinated with bald VLPs. Serum for the hamsters’ terminal bleed was used to neutralize monovalent NiV F/G, HeV F/G, EBOV GP, and the multivalent pseudotyped VSV particles and entry of the virus into the cells was analyzed by Renilla Luciferase assay. The mean neutralization read out for the monovalent and multivalent pseudotyped VSV was calculated and graph plotted using Graphpad software. **B** Monovalent and multivalent pseudotyped VSV were neutralized with different dilutions of sera from hamsters vaccinated with multivalent VLP vaccine. Virus neutralization was done in a similar manner as the negative controls. The average neuralization read outs were calculated and graph derived using Prism Graphpad software. **C** Hamsters were vaccinated with the multivalent pseudotyped VSV. Sample processing was done as for the negative controls. The mean neutralization read out was calculated and graph derived using Prism graphpad software. **D**, **E** Normalized graphs for sera from hamsters vaccinated with the multivalent VLPs and pseudotyped VSV. **F** Forty microliters of VLP and pseudotyped VSV preparations were analyzed by Western blotting to compare incorporation of glycoproteins following observation that pseudotyped VSV was eliciting a relatively stronger immune response compared to VLPs.

Following vaccination, we determined the immunogenic properties of the VLPs in hamsters. We vaccinated five hamsters intramuscularly at six weeks of age using 50 µl (30 µg) of the vaccine preparation in 50 µl of ALUM adjuvant and boosted on days 21 and 42 post first inoculation. Five negative control hamsters were vaccinated with 50 µl of bald VLP preparation in 50 µl of ALUM adjuvant and boosted on days 21 and 42 after first inoculation. The amount of protein inoculated was determined by Bradford assay. Blood samples were collected weekly from day 0 to day 49. Final bleed serum neutralization was determined against fresh batches of pseudotyped VSV virions incorporating NiV F/G only, HeV F/G only, EBOV GP only, or a multivalent VSV virions carrying the three viral glycoproteins using a Renilla Luciferase kit 24 h.p.i.^38^. Supplementary Figs. SF1A to SF1E show graphs of monovalent and multivalent pseudotyped VSV neutralized with different dilutions of antisera from individual negative control hamsters, showing no antibodies against the NiV F/G, HeV F/G, or EBOV GP. Supplementary Figs. SF1F to SF1J show graphs of monovalent and multivalent pseudotyped VSV virions neutralized with different dilutions of sera from individual VLP-vaccinated hamsters. Figures SF1K to SF1O are sigmoidal graphs derived from neutralization read outs for the sera from the individual VLP-vaccinated hamsters. Figure 6A shows the averaged neutralization for all negative control hamsters, while Fig. 6B shows the averaged neutralization trends for sera from hamsters vaccinated with VLPs. Figure 6C is a sigmoidal curve derived from the average neutralization data for sera from hamsters vaccinated with VLPs. The VLP-vaccinated hamsters elicited neutralizing antibodies against all NiV F/G, HeV F/G, and EBOV GP, but were weakest against EBOV.

#### The multivalent pseudotyped VSV virions elicited strong neutralizing antibody responses in hamsters

We also immunized different groups of hamsters with the pseudotyped VSV virions. These groups were treated similarly to the group which was immunized with VLPs. Serum samples were also treated as those collected from hamsters vaccinated with the VLPs. Figures SF2A to SF2E show graphs of monovalent and multivalent pseudotyped VSV neutralized with different dilutions of sera from the multivalent pseudotyped VSV vaccinated hamsters. Figures SF2F to SF2J show sigmoidal graphs derived from neutralization for sera from the pseudotyped VSV vaccinated hamsters. Figure 6D shows the averaged neutralization graphs for antisera derived from hamsters vaccinated with the multivalent pseudotyped VSV vaccine. Figure 6E is a sigmoidal curve derived from the average neutralization read outs for hamsters vaccinated with pseudotyped VSV. The pseudotyped VSV vaccinated hamsters elicited strong neutralizing antibody responses against all NiV F/G, HeV F/G, and EBOV GP. Comparison of Fig. 6C to E illustrates that the neutralizing antibody responses to the multivalent VSV vaccine were stronger than those to the multivalent VLP vaccine, likely due to the higher level of incorporation of the glycoproteins into the VSV virion vaccine per equal amount of sample tested, coincidently produced roughly from equal amounts of cells (Fig. 6F). Because the multivalent pseudotyped VSV preparation had higher incorporation of the glycoproteins (except for NiV F) and it elicited a stronger antibody response, it was subsequently used for the challenge experiments. We also quantified the amount of IgG in the sera for the mock vaccinated hamsters and that from VLPs and pseudotyped VSV vaccinated hamsters using an ELISA. The data shows detection of similar levels of IgG antibodies among all groups indicating that increased neutralization was not due to an increase in total antibodies produced, but to specific neutralizing antibodies (SF 3).

### Multivalent vaccines elicited time-dependent neutralizing antibody responses

**Fig. 7:**
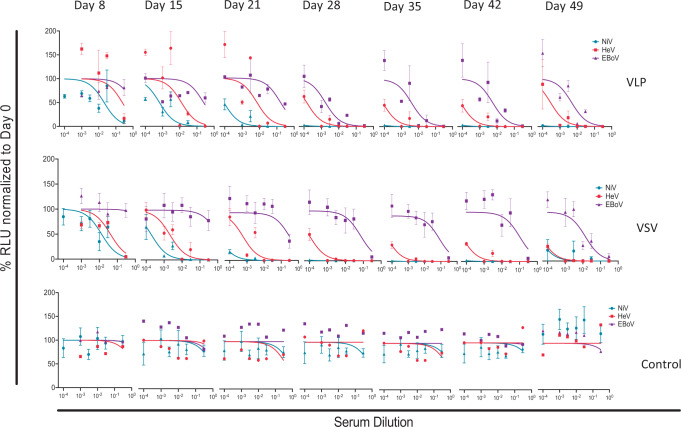
Multivalent pseudotyped VSV and VLPs elicited time-dependent neutralizing antibody titers. Neutralization tests were performed for individual hamsters, and the results shown are averages of individual hamster sera. Sera from pseudotyped VSV and VLP-vaccinated hamsters and control groups were analyzed for eight time points and data normalized to day 0. Error bars represent standard deviation.

After determining the terminal-bleed neutralizing antibody responses to the VLPs and pseudotyped VSV vaccines, we determined the weekly responses starting from vaccination priming, as performed for the terminal bleed samples. Figure 7 shows the averaged results of monovalent pseudotyped VSV for NiV, HeV, and EBOV VSV virions neutralized using the weekly bled sera. The results indicate that immune responses to NiV and HeV develop very early after vaccination priming, while that for EBOV lags behind but becomes stronger a week after vaccination boosting (i.e., day 28). Generally, as expected, neutralizing antibody titers continued to improve thereafter for the testing period.

#### Multivalent pseudotyped VSV protected hamsters against NiV, HeV or EBOV challenge

**Fig. 8:**
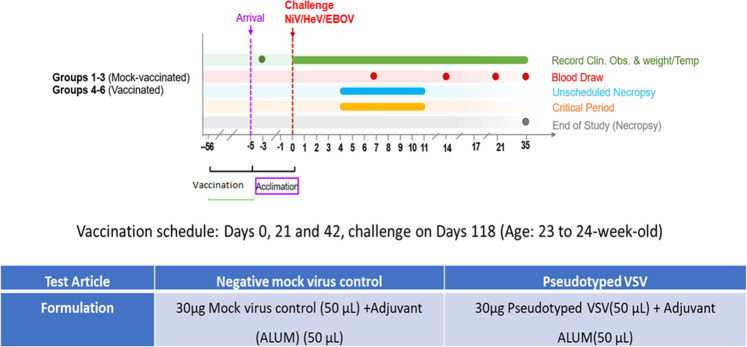
The study design. The figure indicates the vaccination schedule for all hamsters used in this study, the amounts of mock and test vaccines applied, the point of challenge, and the period that the challenge experiment lasted.

We then vaccinated a new batch of hamsters as performed for Fig. 6. The hamsters were grouped into six groups of six hamsters per group. The first eighteen hamsters were mock vaccinated with phosphate-buffered saline (PBS) and Alum, and the second group of eighteen hamsters vaccinated with the multivalent pseudotyped VSV test vaccine (Fig. 8). Sera from the vaccinated hamsters was tested for neutralizing antibodies against NiV, HeV, and EBOV prior to challenge as performed in Fig. 6. The hamsters vaccinated using the multivalent pseudotyped VSV vaccine elicited a strong antibody response (SF 4A, B, and C). Further, sera from these hamsters also neutralized live NiV, HeV, and EBOV (SF 4C, D, and E). In the mock and test vaccine groups, six hamsters were separately challenged with NiV, HeV, or mouse adapted EBOV (maEBOV) five days after arrival at the NIAID Integrated Research BSL-4 laboratory (Fig. 8).

**Fig. 9:**
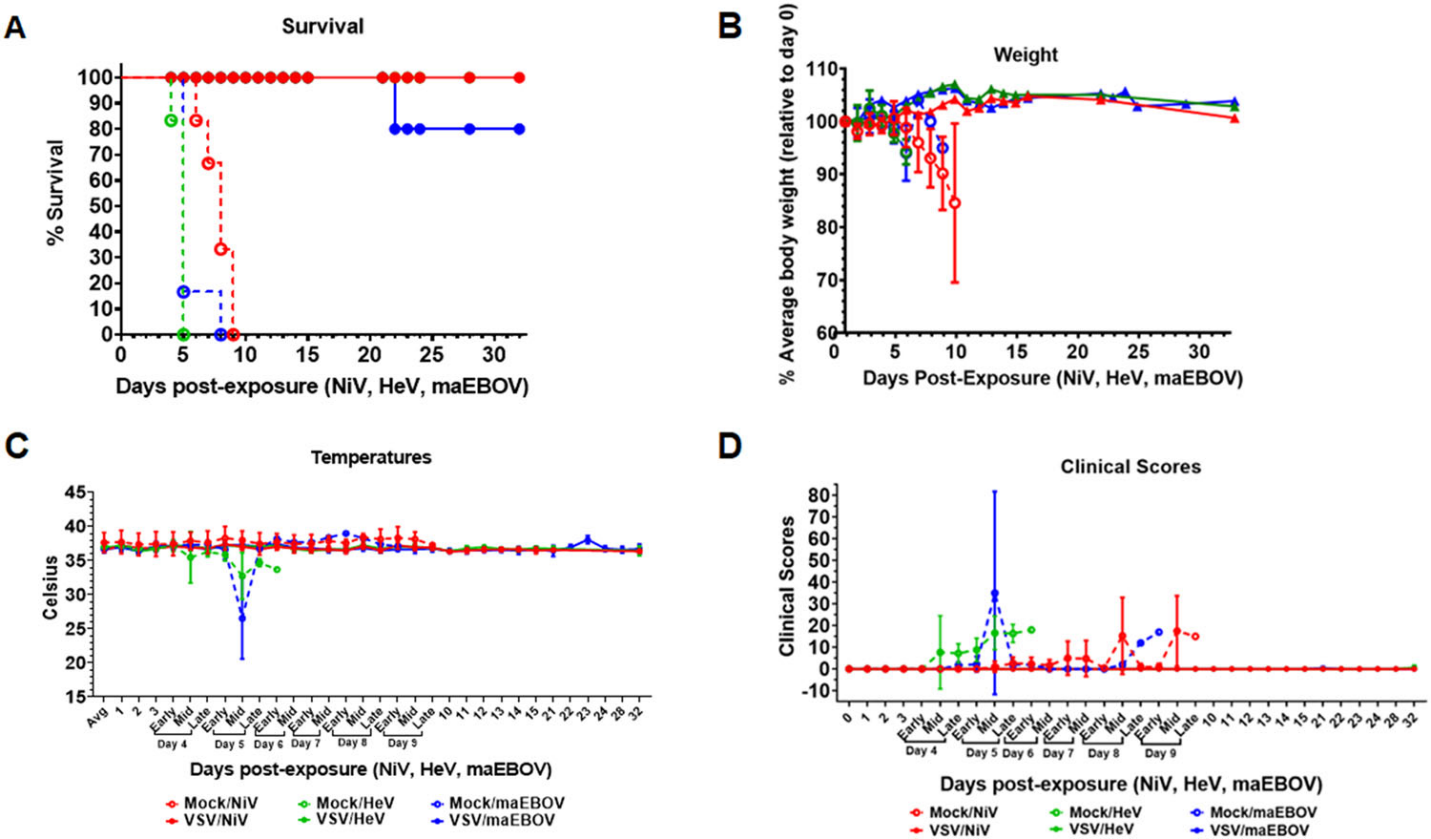
Analysis of hamster survival, weight, and temperatures during the course of challenge experiments. **A** Percent survival of test-vaccinated vs. mock-vaccinated hamsters, following challenge with live NiV, HeV, or maEBOV. Hamsters vaccinated with the test vaccine survived challenge with all the three virulent viruses except that one hamster died from a lymphoma (unrelated cause). Those vaccinated with mock VSV virions were euthanized at critical control points after signs of disease. **B** Weight records for the test vs. mock vaccinated hamsters groups. Hamsters in the test vaccine group showed minimal variation on weight during the course of experiments while those in the mock-vaccinated groups registered significant weight loss prior to euthanasia. **C** Body temperature records for the mock vs. test-vaccinated groups. Hamsters test-vaccinated showed minimal variation in body temperatures while the variation was marked for the mock groups. **D** Clinical scores for the multivalent pseudotyped VSV vaccinated hamsters compared the mock-treated group. Clinical scores were based on a 15-point scale that includes scoring appearance, respiratory signs, mobility, temperature, and neurological signs such as paralysis, seizures, and appearing moribund. Additive scores of 15 or greater required immediate consultation with the veterinarian to determine animal disposition. There was marked deviation of clinical scores between test and mock vaccinated groups. Solid lines represent test-vaccinated animals, while dashed lines represent mock-vaccinated animals.

All hamsters in the test vaccine group survived challenge with the three viruses and exhibited no adverse signs of disease (Fig. 8A), although we lost one maEBOV-challenged hamster to an unrelated lymphoma. All mock vaccinated hamsters had to be euthanized due to severe disease post-challenge (Fig. 9A). Hamsters challenged with NiV had to be euthanized by day 9, those challenged with HeV by day 5, and those challenged with maEBOV by day 8 post-infection (Fig. 9A). There was also a decrease in body weight and temperature for the mock vaccinated group during the course of the experiment as shown in Fig. 9B, C, respectively. During the experiments, the hamsters were also scored clinically based on appearance, respiration, mobility, body temperature, neurological signs, paralysis, seizures, and moribund status. The highest score was 15, which was attained if a hamster was unable to access food or water, had a 4 °C drop in body temperature from the baseline and if it was moribund. Other scores fell between 0 and 15. Hamsters were monitored daily during early, mid, and late hours. All the hamsters vaccinated with the multivalent VSV vaccine did not show any deviation on any of the parameters from the baseline, while all the mock vaccinated hamsters recorded a significant deviation on a number of these parameters prior to euthanasia (Fig. 9D). In summary, these challenge experiments demonstrated that the test multivalent VSV vaccine protected hamsters from challenge from virulent NiV, HeV, and EBOV infections at 100% efficacy and with 100% safety, ~4 months post-vaccination.

#### Multivalent pseudotyped VSV vaccinated hamsters did not suffer pathology following challenge with virulent NiV, HeV, and EBOV

**Fig. 10:**
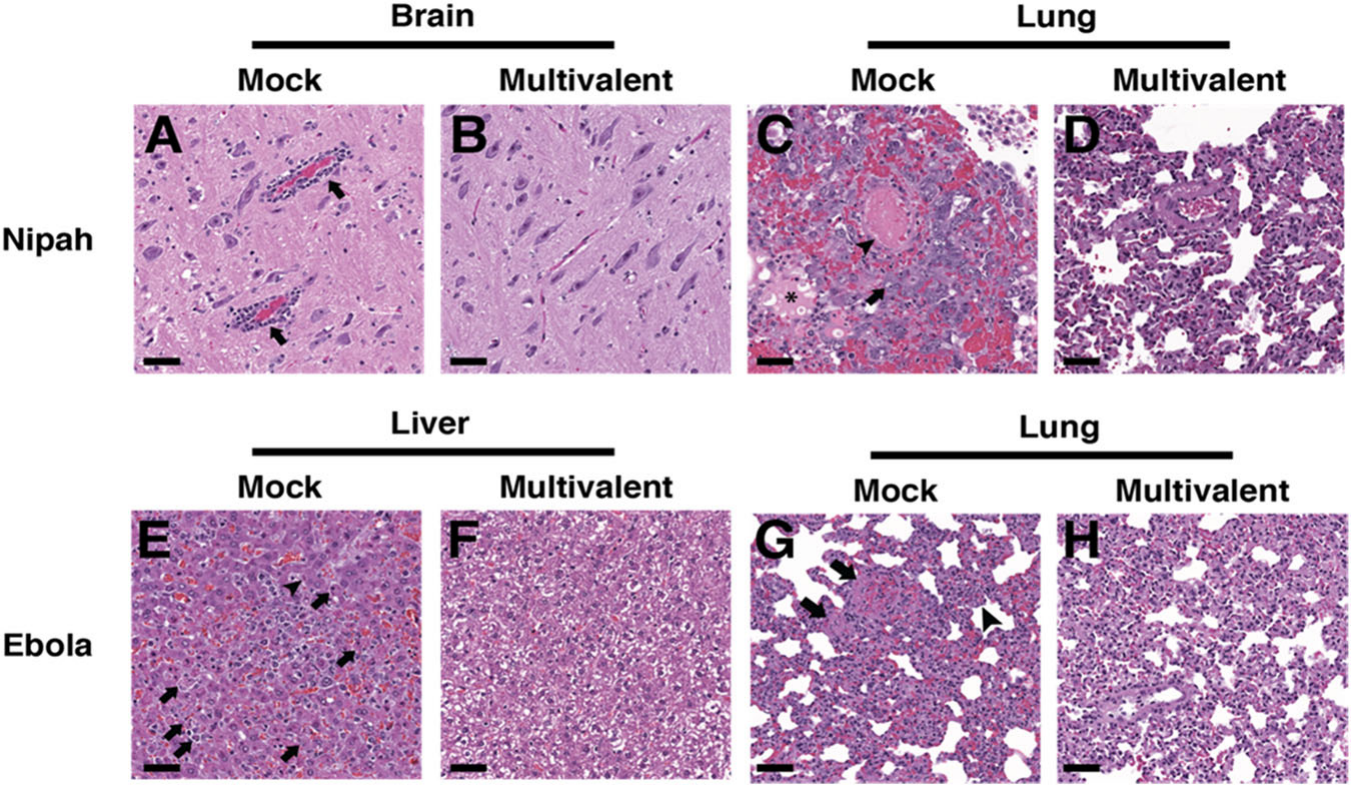
Multivalent VSV virion vaccination prevented the development of histologic lesions in hamsters challenged with Nipah, Hendra, or Ebola viruses. Tissues from representative mock vaccinated (**A**, **C**, **E**, **G**) or multivalent VSV virion vaccination (**B**, **D**, **F**, **H**) following challenge with either Nipah (**A**–**D**) or Ebola virus (**E**–**H**). **A** Brain tissue from a representative mock vaccinated hamster with inflammatory perivascular cuffs (arrows). **C** Lung tissue from a representative mock vaccinated hamster with intravascular fibrin thrombi (arrowhead), edema in alveoli (asterisk), and type II pneumocyte hyperplasia with atypia (arrow). **E** Liver tissue from a from a representative mock vaccinated hamster with lobular hepatitis, hepatocyte necrosis (arrow), and intracytoplasmic viral inclusions (arrowhead). **G** Lung tissue from a representative mock vaccinated hamster with intravascular fibrinocellular debris (arrows) and expansion of alveolar septa with inflammatory cells (arrowhead). Lesions caused by Hendra virus resembled those caused by Nipah virus. H&E, scale bar = 50 μm.

Following euthanasia, different tissues were collected from the challenge hamsters for histopathology. Tissues used for analysis included the brain, liver, and lung. Figure 10A, C demonstrates presence of pathology in the brain and lung, respectively, for mock-vaccinated and NiV challenged hamsters, but not for the multivalent pseudotyped VSV vaccinated and NiV challenged hamsters (Fig. 10B, D). Pathology and results due to NiV challenge were similar to those caused by HeV challenge. Figure 10E, G demonstrates pathology in the liver and lung, respectively, in the mock vaccinated and EBOV challenged hamsters. The multivalent pseudotyped VSV vaccinated and EBOV challenged hamsters did not present pathology (Fig. 10F, H).

#### Sera from the multivalent pseudotyped VSV vaccinated hamsters cross-neutralized pseudotyped VSV incorporating glycoproteins for Cedar virus

Given the successful neutralization of both NiV and HeV, we then asked whether a related henipavirus, Cedar virus (CedV), with moderate sequence similarities to NiV and HeV G and F (SF 5A) may also be neutralizable with the vaccinated sera. We first tested the binding levels of sera from the vaccinated multivalent VSV hamsters on HEK293T cells expressing the surface G or F glycoproteins from either NiV or CedV. Interestingly, we observed that the sera bound relatively well particularly to the F proteins (SF 5B). Since F is relatively more conserved than G (SF 4A), this raises the possibility of serum cross-neutralization of other henipaviruses. We thus tested one serum with particularly good F binding levels (serum 6.2) for neutralization of pseudotyped CedV. At a 1:30 dilution of this serum, we observed neutralization of pseudotyped CedV (SF 5C). Altogether, these results suggest that the multivalent vaccination strategy is capable of yielding cross-protective antibodies against henipaviruses beyond NiV and HeV, justifying further use and optimization of this vaccination strategy towards the goal of broadly protective vaccines.

### CD4^+^ T cell depletion during vaccination prevents CD4^+^ and CD8^+^ cell restimulation to vaccine antigens

To assess whether T cells respond to viral antigens after vaccination, we harvested splenocytes from CD4^+^ T cell-depleted or isotype antibody control treated hamsters 3 weeks after delivery of multivalent VSV intramuscular vaccinations (SF 6). Notably, while CD8^+^ T cell depletion was not effective in hamsters using available anti-CD8 antibodies, circulating CD4^+^ T cells were depleted to less than 1% at the time of vaccination, and slowly rebounded to an average of ~35% of control animal CD4^+^ T cell counts by day 20 (SF7). Splenocytes were stained with dye that can report on proliferation, cultured for 5 days in the presence of media alone, pseudotyped bald virus control (PBC), monovalent NiV F/G (NiV), HeV F/G (HeV), EBoV GP (EBoV), or multivalent pseudotyped viruses, or with ConA stimulation as a positive control (SF 8). Cells were then harvested and stained for flow cytometry analysis to determine proliferated cells (gating strategy in SF 8). Proliferation of CD4^+^ (SF 9A) and CD8^+^ (SF 9B) T cells were compared between the isotype control and the CD4^+^ T cell-depleted hamsters. CD4^+^ and CD8^+^ T cells of both groups showed robust capacity for proliferation following stimulation with the ConA as a positive control (expected since that by day 20, CD4^+^ T cell counts in the CD4^+^ T cell depleted animals had rebounded to an average ~35% of control animals). Due to a variation in cell toxicity of each pseudotyped virus, we could not compare across pseudotyped virus treatments, only between isotype and CD4^+^ T cell-depleted hamster groups within each activation treatment. Interestingly, all pseudotyped viruses induced proliferation of both CD4^+^ and CD8^+^ T cells from the isotype antibody control treated hamsters. However, in almost all cases, cells from the CD4^+^ T cell-depleted hamsters were unable to proliferate in response to the pseudotyped viruses. These data indicate that some form of T cell immune memory is generated by immunization with the multivalent VSV pseudotyped vaccines, and that the presence of CD4^+^ T cells *at the time of vaccination* is necessary for adequate vaccine antigen restimulation of CD4^+^ and CD8 T^+^ cells three weeks later.

### CD4^+^ T cell depletion did not significantly change neutralizing antibody production

CD4^+^ cells were depleted from hamsters^39^ with an intraperitoneal antibody injection 24 h prior to vaccination intramuscularly with multivalent VSV pseudotyped viruses. Blood was collected weekly post vaccination, and serum from the isotype control or CD4^+^ T cell-depleted hamsters was evaluated for ability to neutralize monovalent NiV F/G, HeV F/G, or EBoV GP pseudotyped viral entry into cells. Viral entry was quantified by measuring Renilla Luciferase activity, normalized to levels from pre-vaccination serum. Despite slight trends of decreased neutralization activity from the CD4^+^ T cell-depleted hamsters, there was no statistically significant difference in neutralizing antibody titers between the two groups (SF 10).

## Discussion

By transfecting and/or infecting HEK 293T cells using plasmids carrying genes for glycoproteins from NiV, HeV and EBOV, we made replication-incompetent multivalent VLPs or pseudotyped VSV incorporating the target glycoproteins on their surface, for application as multivalent vaccines. These particles showed a good incorporation of the glycoproteins on their surface, were biologically active, and induced a neutralizing antibody response in hamsters. VLPs and replication-competent pseudotyped VSV virions have been used in vaccine studies to deliver target viral proteins in their native form^31,39–42^.

In this study, we found a higher incorporation of the target glycoproteins onto the pseudotyped VSV when compared to VLPs by Western blot analysis (Fig. 6F). This could be attributed to the pseudotyped virus particles acquiring more of the glycoproteins on their surface as they bud, in addition to having a slightly larger surface area for protein acquisition compared to the NiV-based VLPs. In the hamsters, although we injected equal amounts of total protein for VSV and VLPs, we noticed a stronger immune response to the pseudotyped VSV particles compared to the VLPs. This may have solely been due to the slightly higher levels of incorporation into the VSV virions compared to the VLPs (Fig. 6F), however, this demands further investigation. As examples of potential explanations, the presence of RNA or other proteins in the VSV particles may improve the immune responses, or the processing of the two particles in cells of the immune system may differ.

Vaccine studies for NiV, HeV, and EBOV have previously been conducted^5,9^. A soluble G protein vaccine for HeV is currently available for use in horses^5^, and a replication-competent VSV-based EBOV vaccine was recently licensed for human use^11,27,28^. However, there is no licensed NiV and HeV vaccine for use in humans. Although studies on individual virus vaccines have been conducted, to our knowledge this is the first study in which the target proteins of different viruses are incorporated onto VLPs or replication-incompetent pseudotyped VSV particles, and also where these two systems are compared. The glycoproteins incorporated onto the VLPs and the pseudotyped VSV virions were analyzed by Western blot analysis, flow virometry, and electron microscopy. The resulting VLPs and pseudotyped VSV virions have the advantage over other systems of presenting the target proteins in their native conformation, allowing for effective adaptive immune responses. These proteins were dense, repetitive, and ordered on the particle surfaces as shown in Figs. 2F, 3F, which conforms to earlier observations for NiV glycoproteins^43^.

In this study, we lyophilized the pseudotyped VSV particles, incorporating the target proteins to determine the effect of temperature on the lyophilized and non-lyophilized particles. This is important because the pseudotyped VSV virions may be vaccines used in high-temperature environments, especially in developing countries which have insufficient cold-chain infrastructure. The lyophilized particles were viable for up to five weeks at 37 °C and lost no viability at 25 °C for the six weeks exposure period (Fig. 5D). Virus vaccines have been lyophilized in the past, although this is not always possible while preserving vaccine efficacy. For example, in Ethiopia, the attenuated PPR vaccine lyophilized using trehalose lost 2 log titer when exposed to 37 °C for 4 days^37^. In our study, the lyophilized pseudotyped VSV lost one log in relative light units when exposed to 37 °C for 7 days, but lost basically no activity for 5 weeks at 37 °C when lyophilized in 5% trehalose. Thus, importantly our methodology may be widely spread for vaccine preservation. This is important when administering the potential vaccine in areas with cold-chain infrastructure challenges, where some of the viruses under study have been detected, such as Bangladesh and various countries in Africa.

The multivalent pseudotyped VSV vaccine incorporating NiV, HeV, and EBOV glycoproteins protected 100% of hamsters from challenge with NiV, HeV, or mouse adapted EBOV (maEBOV) and the protection co-related with neutralizing antibody levels. Individual NiV, HeV, or EBOV vaccines have been investigated previously. A recombinant G glycoprotein subunit vaccine for NiV protected ferrets for 12 months post vaccination^29^. Other NiV vaccines trials have been conducted as well as for HeV^44–46^, and a number of virus-vectored EBOV vaccines have been developed^47–54^. Our multivalent vaccine approach may have practical impacts where administration of multiple individual vaccines is less than practical, and may cut cost, particularly where there is overlap of these viral diseases or their reservoir hosts, increasing the potential of future pandemics for these or related viruses. In Africa for instance, similar methodologies may be preferred for the following viruses with similar distribution patterns in the Northern, Eastern and Central parts of the continent: Rift valley fever virus (RVFV), Peste des petits ruminants virus (PPRV), Lumpy skin disease virus (LSDV), Sheep pox virus (SPPV) and Goat pox virus (GTPV). Foot and mouth disease virus (FMDV) is also widely distributed in the Eastern and Central Africa^55–58^, and there is increasing serological evidence of the Middle East Respiratory Syndrome Coronavirus (MERS-CoV) in the Middle East, Northern and Eastern Africa^59^. The multivalent approach has huge potential to combat both animal and human diseases and zoonotic episodes.

In this study, it is also important to note that we used immunogenic proteins from henipaviruses, which are unrelated to EBOV. EBOV is a filovirus with a different set of genetic make-up compared to the henipaviruses, which are paramyxoviruses. We demonstrated that immunogenic proteins from unrelated viral families can be combined into similar replication-incompetent viral particle platforms. This approach will enable production of combined vaccines hence reducing the logistics of production and cost compared to monovalent vaccines. An added benefit is that a multivalent vaccine may increase the breadth of vaccine protection. For example, targeting NiV and HeV glycoproteins simultaneously may lead to eliciting neutralizing antibodies against other henipaviruses due to the focusing of the immune response to conserved henipaviral epitopes. Evidently, the neutralizing antibodies responses of most prior individual henipaviral vaccine attempts focused on the relatively variable G glycoprotein. However, as shown in SF4, we observed multivalent serum antibody binding primarily to the more conserved CedV F, suggesting that the mechanism of neutralization observed is primarily F-mediated. Further, there was partial neutralization of pseudotyped CedV virions. Thus, we speculate that the multivalent NiV-HeV-EBOV vaccine may partially protect against Cedar virus (CedV) and possibly other related henipaviruses, constituting an additional potential benefit of our multivalent viral-particle vaccination strategy. We also speculate that the multiple conserved structural elements of the class I fusion proteins NiV F, HeV F, and EboV GP may be responsible for the broadly binding and neutralizing antibodies elicited by our multivalent VSV vaccine.

Whereas the T cells that proliferated in response to pseudotyped virus (SF 9) are likely memory T cells, we are currently unable to fully characterize all responding immune cells due to lack of appropriate immunological reagents for use in the hamster. It was interesting that even the pseudotyped bald virus control caused proliferation of the isotype-treated hamster CD4^+^ and CD8^+^ T cells. This is likely due to the VSV pseudotyped virus vaccine carrying its own antigens that result in the development of antigen-specific memory T cell formation. Therefore, memory T cells of these animals would be able to respond to VSV itself, or NiV, HeV, or EBoV upon later infections of those animals, although protection against VSV remains to be determined. It is also likely that some B cell memory populations and antibodies produced after vaccination would be reactive to VSV antigens. The T cell proliferation assay used in this work is not able to differentiate between NiV, HeV, or EboV-specific responses, from baseline VSV-specific T cell responses since they all share VSV antigens. However, future testing could investigate reactivation against recombinant specific antigen stimulation from these other viruses.

The minimal effect of CD4^+^ T cell depletion on neutralizing antibody production from a single vaccination is not completely surprising, as some antigens are able to induce antibody responses that are not dependent on CD4^+^ T cell help. In addition, it is possible that certain tissue resident T cells may remain after a single administration of antibody depletion, therefore, additional antibody depletion doses along with additional vaccine boosts may yield a larger disparity in the neutralizing antibody production, or on the affinity and/or isotype of the resultant antibody generated under these conditions. Still, it could be possible that CD4^+^ T cells are not as important for neutralizing antibody production from this type of vaccination. Ko et al. demonstrated that different vaccine adjuvants may or may not require CD4^+^ T cells for adequate antibody production or isotype class switching in mice^60^. This may apply to certain infections as well, whereby CD4^+^ T cell-depletion did not affect antibody responses after Dengue virus infection^61^. Overall, the presence of CD4^+^ T cells at the time of vaccination contribute to CD4^+^ and CD8^+^ T cell memory formation, but appear to be less important for neutralizing antibody production after a single immunization. Ultimately, the high efficacy of the multivalent NiV/HeV/EboV vaccine correlates well with neutralizing antibody responses.

## Methods

### Cell cultures

The cells used in this study were sourced from ATCC and used below their 20th passage. Cells were tested to be mycoplasma free. The human embryonic kidney 293T cells and Vero cells were grown in Dulbecco’s minimum essential medium supplemented with 10% fetal bovine serum (FBS) and 1% penicillin/streptomycin. The cells were maintained in this medium throughout the study.

### Protein expression vectors

Expression plasmid for NiV M, HeV F, and EBOV GP was pCAGGS while NiV F/G and HeV G were in pCDNA3.1. The protein constructs had the following DNA tags: NiV M–flag, NiV F–flag, NiV G–myc, HeV F–AU1, HeV G–HA, and EBOV GP–V5.

### Cell transfections

Human embryonic kidney (HEK) 293T cells were grown in Dulbecco’s complete medium to a 80–90% confluent cell monolayers. The cells were transfected with the plasmid constructs for 8 h using Lipofectamine 2000 (Invitrogen Inc) according to the manufacturer’s guidelines. After 48 h post-transfection, the VLP-containing cell supernatants (SUP) were harvested for concentration and purification of the VLPs. For the pseudotyped VSV preparations, the cells were infected with 1:10,000 dilution of VSVΔG after 12–14 h following transfection. The pseudotyped VSV’s and VLPs’ SUPs were harvested at the same time. The cells were lysed using RIPA buffer to determine protein content in the lysates and also stained with primary and secondary antibodies to determine protein cell surface expression via flow cytometry.

### VLPs harvesting and purification

VLPs and pseudotyped VSV released in the transfected cell supernatants were harvested and clarified by centrifugation at 2200 rpm for 10 min at 4 °C. The clarified SUPs were concentrated by ultracentrifugation through 20% sucrose cushion in TN buffer (0.1 M NaCl; 0.05 M Tris-HCL, pH 7.4) at 110,000 × *g* for 1.5 h at 4 °C. The resulting VLP and pseudotyped VSV pellet was resuspended in endotoxin-free 5% sucrose buffer and stored at 4 °C for short-term use or −80 °C for extended storage. 293T cells were also transfected with empty pCDNA3.1 and pCAGGS plasmids and their supernatants similarly processed to be used as negative controls.

### Protein determination

The total protein concentration of the purified the VLPs and pseudotyped VSV preparations was measured using the BCA (Bicinchoninic acid) method following the manufacturer’s instructions (Thermo Scientific Laboratories).

### Western blot analysis

The collected VLPs and pseudotyped VSV virions were analyzed by Western blotting to determine incorporation of glycoproteins. 10 µl of 6× SDS-PAGE dye were added to each sample, individually loaded onto each lane of a polyacrylamide gel and ran at 100 V for 2 h. The proteins were transferred onto a nitrocellulose membrane at 0.5 A for 1.5 h. Glycoproteins NiV M and NiV F were blotted in 1:500 dilution of mouse anti-flag (Sigma Cat #A2220), EBOV GP in 1:500 mouse anti-V5 (Invitrogen Cat # MA5-15253), while NiV G, HeV F, and HeVG were blotted in 1:1000 dilution of rabbit anti-Myc (sigma Cat # PLA0001), anti-AU1 (Invitrogen Cat # A190-125A) and anti-HA (Biolegend Cat # 923501) primary antibodies, respectively. We used Alexa Fluor 647 goat anti-mouse IgG (Invitrogen Cat # A21236) and Alexa Fluor 488 goat anti-rabbit IgG (Invitrogen Cat # A21244) fluorescent secondary antibodies at a 1:1000 dilution. The Western blots, including molecular markers, for the detection of Pseudotyped VSV or particles or VLPs and lysates are shown in Supplementary Fig. 11. All Western blots were protein levels are compared were derived from same experiment and were processed in parallel.

### Flow virometry and cytometry

VLP and pseudotyped VSV samples were stained with primary mouse anti-flag, anti-V5, and rabbit anti-MyC, anti-AU1, and anti-HA antibodies at a dilution of 1:200 dilution for 1 h at 4 °C. The samples were then washed with FACs buffer (phosphate-buffered saline with 1% fetal bovine serum) by ultracentrifuging twice at 110,000 × *g* for 1 h at 4 °C. Goat anti-rabbit 647 and goat anti-mouse 488 fluorescent secondary antibodies (Invitrogen^R^) were then added also at 1:200 dilution and allowed to bind at 4 °C for 30 min. The samples were washed once in FACs buffer by spinning at 110,000 × *g* and resuspended in PBS with 0.5% paraformaldehyde (PFA). The surface proteins were detected using a Guava easyCyte 8HT flow cytometer in which the forward and side scatter (FSC and SSC) settings were slightly modified to detect smaller particles. The relatively small viral particles were differentiated from suspension buffer debris by gating in the forward versus side scatter plot. The transfected 293T cells were collected and stained in a similar manner as VLPs, but were spun 3 times at each stage at 2200 rpm in a 96-well plate. They were also resuspended in PBS with 0.5% PFA and the protein cell surface expression (CSE) determined using the Guava easyCyte 8HT flow cytometer. Antibody catalog numbers are as indicated for Western blotting.

### Viral infectivity assay

Vero cells at 40% confluency were infected with the pseudotyped VSV at 1:100 to 1:1,000,000 dilutions and incubated for 24 h. The cells were lysed and mean luminescence was taken using Renilla Luciferase assay as per manufacturer’s instructions (Promega^R^).

### Lyophilization protocol

Negative plasmid pseudotyped VSV and NiV G only pseudotyped VSV served as the negative controls. The controls and the multivalent VSV were serially diluted to 1:100, 1:1000, 1:10,000, 1:100,000, and 1:1,000,000 in 5% trehalose. Tubes containing the diluted samples were immersed in liquid nitrogen for 1 min and transferred onto lyophilizing jars. Lyophilizing was run for 24 h. The lyophilized pseudotyped virus was stored at 4 °C in a vacuum jar until required.

### Electron microscopy

VLPs and the pseudotyped VSV were collected and purified as previously described. The virions were adsorbed on a Formvar carbon-coated copper grid by floating it on a drop of sample suspension for 15 min and then fixing using 2% formaldehyde/2% glutaraldehyde solution in 0.1 M cacodylate buffer. The grids were blotted, and then negatively stained with 1% aqueous uranyl acetate and viewed using a FEI T20 electron microscope.

### Protocol for immunizing hamsters

Animal studies were approved by the Institutional Animal Care and Use Committee (IACUC) (Cornell Protocol number 2018-0063). Fifteen five weeks old female hamsters (Charles River Laboratories) were housed in cages for two weeks in the East Campus Research Facility (ECRF), Cornell University, before starting the immunization protocol experiments. Five hamsters per treatment were immunized intramuscularly with 50 µl of the test sample in 50 µl of ALUM as follows; group one was injected with the empty plasmid controls, group two with the VLPs and group three with the pseudotyped VSV. The total protein in the test vaccines was 30 µg determined using the BCA method. The hamsters were given a vaccine boost on days 21 and 42. They were bled on days 0, 7,14, 21, 28, 35, 42, and terminated on day 49 and euthanized.

### In vivo CD4^+^ T cell depletion

When indicated, anti-mouse CD4 or anti-mouse CD8 antibodies were administered in an attempt to deplete these T cell subsets in the experimental hamsters prior to vaccination. While the anti-mouse CD4 antibody was able to deplete the hamster CD4^+^ T cells, the CD8^+^ T-cell depletion was unsuccessful. For CD4^+^ T cell depletion, hamsters were intraperitoneally injected with 1 mg of either isotype control (cat# BP0090) or anti-mouse CD4 (cat# BP0003-1; BioXcell, Lebanon, NH) antibodies one day prior to vaccination. Blood was extracted 24 h and weekly post-depletion to assess CD4^+^ T cell levels in circulation (SF7).

### Ex vivo splenocyte activation

After hamster euthanasia on day 20 post vaccination, spleens were extracted, mechanically digested, and filtered through 70 mm filters. Red blood cells were lysed with ACK lysis buffer, and splenocytes were stained with 5 μM CFSE as per manufacturer’s instructions (cat# C34554; Thermo Fisher Scientific). Next, cells were seeded into 24-well plates in media (RPMI 1640 medium with 10% FBS, 4 mM L-glutamine, 0.1 mM nonessential amino acids, 1 mM sodium pyruvate, 100 U/ml penicillin and streptomycin). Cells were left untreated or treated with the indicated pseudovirus, or with 1 μM Concanavalin A (ConA) (cat# J61221.MC; Thermo Fisher Scientific) for 5 days. Cultured hamster splenocytes were stained with anti-CD4-APC/Fire750 (clone GK1.5; BioLegend) and anti-CD8-PE (clone 341; Invitrogen) antibodies simultaneously with eBioscience Fixable Viability Dye-efluor-506. Cells were washed and then fixed with 1% paraformaldehyde and ran on the Thermo Fisher Attune NxT, and analyzed with FlowJo Software, V10 (SF8, SF9).

### Serum neutralization

Serum samples from both the VLP and pseudotyped VSV vaccinated hamsters were diluted 1:10, 1:30, 1:100, 1:300, 1:1000, 1:3000, 1:10,000, and 1:30,000. NiV F/G pseudotyped VSV was diluted 1:10,000, the HeV F/G pseudotyped virus 1:1000, the EBOV GP pseudotyped VSV 1:100 and the multivalent pseudotyped VSV 1:10,000 as previously determined (Fig. 3A). Each pseudotyped VSV was dispensed onto microcentrifuge tubes and equal amounts of each serum dilution added. They were incubated for one hour at 37 °C in a shaker and then 100 µl dispensed onto Vero cells at 40% confluency in duplicates. Neutralization of the different pseudotyped VSV was measured using the viral infectivity assay.

### Fluorescence neutralization assay 50 (FRNA50)

All assays were run on irradiated and heat-inactivated sera. VeroE6 (BEI #NR596) cells were seeded at 3 × 10^4^ in 100 µL DMEM + 10% FBS in 96-well Operetta plates (Greiner Bio-One). The following day, a series of twelve-point dilutions, each 1:2, was performed in duplicates (1:20, 1:40, 1:60, etc.) in 96-well 1.2 mL cluster tubes (Corning). Starting dilution depended on the virus, for Hendra virus (HeV) and Nipah virus (NiV), starting dilution was 1:40, for mouse adapted Ebola virus (maEBOV), the starting dilution was 1:20. Then, stock Hendra virus, Nipah virus and mouse adapted Ebola virus was diluted in serum free media and was added to the sera in each cluster tube at 0.5 multiplicity of infection (MOI) for HeV, 1.0 MOI for maEBOV 0.1 MOI for NiV using a liquidator, doubling the total volume in each well and further diluting sera 1:2. Thus, the final starting dilution was 1:80 and 1:40. The sera/virus mixture was then mixed by pipetting up and down with the liquidator and incubated for 1 h 37 °C/5% CO_2_. Assay was performed based on the methods previously described in ref. ^62^. After the sera/virus mixtures was added to the plates, plates were incubated for 24 h. For the fluorescence staining, the primary antibody was HeV Ab Mix-PA8903&8904 Termination (IBT) prepared at 1:2000, Mouse antibody, EBOV VP40 BMD04B007 A11 (USAMRIID) prepared at 1:2000 and Rabbit Ab NIV PA8905 Terminal (ThermoFisher) at 1:2000 in blocking buffer at room temperature. Plates were incubated with primary antibody for 60 min on a rocker. The secondary antibody was Goat α-rabbit IgG (H + L), Alexa Fluor 594 Conjugate (Life Technologies) prepared at 1:2500 in 1X PBS. Plates were incubated with secondary antibody at room temperature for 30 min on a rocker and in the dark. The fluorescence intensity of a sample at each dilution was compared to the FRNA50 values, and the lowest dilution that is equal to or less than the FRNA50 value was recorded.

### Antibody binding assay

10 cm plates of HEK293T cells were transfected with 15 µg of NiV G, NiV F, CedV G, or CedV F or negative control pCAGGS vector expression plasmids in the presence of PEI (1 mg/mL) at a 4:1 transfection to plasmid ratio. After 24 h, cells were incubated with hamster serum for 30 min on ice. Serum samples from Mock or VSV vaccinated hamsters were diluted 1:30 in PBS with 1% BCS blocker prior to incubation with cells. Cells were washed 3× (300 × *g*, 5 min, 4 °C) with cold PBS. Goat anti-hamster secondary antibody (1:5000) was added to the cells and left to bind for 30 min on ice prior to washing and detection by flow cytometry.

### Protocol for challenge experiments

A new batch of 36 hamsters was housed at the Cornell University ECRF. Eighteen hamsters were vaccinated intramuscularly with 50 µl (30 µg) of the multivalent pseudotyped VSV in 50 µl of ALUM. Eighteen hamsters in the negative control group were given 50 µl (30 µg) of mock virus in 50 µl of ALUM. Hamsters were at ECRF for 113 days before transfer to National Institute of Allergy and Infectious Diseases (NIAID) Integrated Research Facility (IRF). At the IRF, the hamsters were allowed to acclimatize for 5 days before onset of challenge experiments. Challenge experiments were done under animal protocol number IRF-022E. The mock and test vaccine hamsters were separated into six treatment groups of six hamsters per group and challenge virus was introduced intraperitoneal. In the mock and pseudotyped VSV vaccinated clusters, six hamsters were challenged with Malaysian strain NiV (19,680 PFU), six with HeV (10,020 PFU), and six with mouse adapted EBOV—maEBOV (10,900 PFU). The approximate LD50 for the challenge viruses was 3280 pfu for NiV, 850 pfu for HeV, and above 110 pfu for ma-EBOV. The higher challenge doses were considered based on the age of hamsters. The study period was 35 days.

### Histopathology

Lung, brain, and liver tissue samples were fixed with 4% paraformaldehyde, paraffin embedded and cut into 3.5-µm sections. The tissue sections were stained with H&E.

### Data analysis

All data were graphed and analyzed by the indicated test using GraphPad Prism Software (San Diego, CA) (*p < 0.05, **p < 0.01, ***p < 0.001, ****p < 0.0001).

### Reporting summary

Further information on research design is available in the Nature Research Reporting Summary linked to this article.

## Supplementary information

Supplemental Information

REPORTING SUMMARY

## Data Availability

All data generated or analyzed during this study are included in this publication and its supplementary information files.
