# Supplementary material for: Multivalent viral particles elicit safe and efficient immunoprotection against Nipah Hendra and Ebola viruses
Source: NPJ Vaccines. 2022 Dec 17;7:166. doi: 10.1038/s41541-022-00588-5 (PMC9759047; doi:10.1038/s41541-022-00588-5)
Supplement: Supplementary file 1 — Supplemental Information [file 41541_2022_588_MOESM1_ESM.docx]

Supplementary Materials for

**Title:**

**Multivalent viral particles elicit safe and efficient immunoprotection against Nipah Hendra and Ebola viruses**

**Authors**:

Duncan G. Ithinji^1,2^, David W. Buchholz^3*^, Shahrzad Ezzatpour^3*^, I. Abrrey Monreal^3*^, Yu Cong^4^, Julie Sahler^3^, Amandip Singh Bangar^3^, Brian Imbiakha^3^, Viraj Upadhye^3^, Janie Liang^4^, Andrew Ma^3^, Birgit Bradel-Tretheway^1^, Benjamin Kaza^3^, Yao Yu Yeo^3^, Eun Jin Choi^3^, Gunner P. Johnston^3^, Louis Huzella^4^, Erin Kollins^4^, Saurabh Dixit^4^, Shuiqing Yu^4^, Elena Postnikova^4^, Victoria Ortega^3^, Avery August^3^, Michael R. Holbrook^4^, and Hector C. Aguilar^3 t^

**Affiliations:**

1. School for Global Animal Health, Washington State University, Pullman, WA, USA

2. Kenya Agricultural and Livestock Research Organization, Nairobi, Kenya

3. Dept. of Microbiology and Immunology, Cornell University, Ithaca, NY, USA

4. National Institute of Allergy and Infectious Diseases (NIAID) Integrated Research Facility, Ft Detrick, Frederick, MD 21702 USA

^t^ **Correspondence to:** [ha363@cornell.edu](mailto:ha363@cornell.edu) (H.C. Aguilar)

*Equally contributing authors

**Methods**

**Serum neutralization**

Serum samples from both the VLP and pseudotyped VSV vaccinated hamsters were diluted 1:10, 1:30, 1:100, 1:300, 1:1000, 1:3000, 1:10000, and 1:30,000. NiV F/G pseudotyped VSV was diluted 1:10000, the HeV F/G pseudotyped virus 1:1000, the EBOV GP pseudotyped VSV 1:100 and the multivalent pseudotyped VSV 1:10000 as previously determined (figure 3A). Each pseudotyped VSV was dispensed onto microcentrifuge tubes and equal amounts of each serum dilution added. They were incubated for one hour at 37^o^C in a shaker and then 100µl dispensed onto Vero cells at 40% confluency in duplicates. Neutralization of the different pseudotyped VSV was measured using the viral infectivity assay.

**Viral Infectivity assay**

Vero cells at 40% confluency were infected with the various pseudotyped VSV at 1:100 to 1:1000000 dilutions and incubated for 24 hours. The cells were lysed and mean luminescence was taken using Renilla Luciferase assay as per manufacturer’s instructions (Promega^R^).

**Fluorescence Neutralization Assay 50 (FRNA50)**

All assays were run on irradiated and heat-inactivated sera. Irradiation and heat-inactivation were performed as described in the “Serology” methods section. VeroE6 (BEI #NR596) cells were seeded at 3x10^4^ in 100 µL DMEM+10% FBS in 96 well Operetta plates (Greiner Bio-One). The following day, a series of twelve-point dilutions, each 1:2, was performed in duplicates (1:20, 1:40, 1:60, etc.) in 96 well 1.2 mL cluster tubes (Corning). Starting dilution depended on the virus, for Hendra virus (HeV) and Nipah virus (NiV), starting dilution was 1:40, for mouse adapted Ebola virus (maEBOV), the starting dilution was 1:20. Then, stock Hendra virus, Nipah virus and mouse adapted Ebola virus was diluted in serum free media and was added to the sera in each cluster tube at 0.5 multiplicity of infection (MOI) for HeV, 1.0 MOI for maEBOV 0.1 MOI for NiV using a liquidator, doubling the total volume in each well and further diluting sera 1:2. Thus, the final starting dilution was 1:80 and 1:40. The sera/virus mixture was then mixed by pipetting up and down with the liquidator and incubated for 1 hour 37°C/5% CO_2_. Assay was performed in accordance to the methods described in “Scalable, semi-automated fluorescence reduction neutralization assay for qualitative assessment for Ebola virus-neutralizing antibodies in human clinical samples”. After the sera/virus mixtures was added to the plates, plates were incubated for 24 hours. For the fluorescence staining, the primary antibody was HeV Ab Mix-PA8903&8904 Termination (IBT) prepared at 1:2000, Mouse antibody, EBOV VP40 BMD04B007 A11 (USAMRIID) prepared at 1:2000 and Rabbit Ab NIV PA8905 Terminal (ThermoFisher) at 1:2000 in blocking buffer at room temperature. Plates were incubated with primary antibody for 60 minutes on a rocker. The secondary antibody was Goat α-rabbit IgG (H+L), Alexa Fluor 594 Conjugate (Life Technologies) prepared at 1:2500 in 1X PBS. Plates were incubated with secondary antibody at room temperature for 30 minutes on a rocker and in the dark. The fluorescence intensity of a sample at each dilution was compared to the FRNA50 values, and the lowest dilution that is equal to or less than the FRNA50 value was recorded.

**Antibody Binding Assay**

10 cm plates of HEK293T cells were transfected with 15ug of NiV-G, NiV-F, CedV-G, or CedV-F plasmid with PEI (1mg/mL) at a 4:1 transfection to plasmid ratio. After 24 hours, cells were incubated with hamster serum for 30 minutes on ice. Serum samples from Mock or VSV vaccinated hamsters were diluted 1:30 in PBS with 1% BCS prior to incubation. Cells were washed 3X (300xg, 5 minutes, 4C) with cold PBS. Goat anti-hamster secondary antibody (1:5,000) was added to the cells and left to bind for 30 minutes on ice. Signals were detected by flow cytometry.

***In vivo* CD4^+^ T cell depletion**

Adapted from published work (39) hamsters were intraperitoneally injected with 1 mg of either isotype control (cat# BP0090) or anti-mouse CD4 (cat# BP0003-1; BioXcell, Lebanon, NH) antibodies one day prior to vaccination. Blood was extracted 24 hours, and then weekly, post depletion to assess CD4^+^ T cell levels in circulation (SF 7).

***Ex vivo* splenocyte activation**

After hamster euthanasia on day 20 post vaccination, spleens were extracted, mechanically digested, and filtered through 70mm filters. Red blood cells were lysed with ACK lysis buffer, and splenocytes were stained with 5μM CFSE as per manufacturer’s instructions (cat# C34554; Thermo Fisher Scientific). Next, cells were seeded into 24 well plates in media (RPMI 1640 medium with 10% FBS, 4 mM L-glutamine, 0.1 mM nonessential amino acids, 1 mM sodium pyruvate, 100 U/ml penicillin and streptomycin). Cells were left untreated or treated with the indicated pseudovirus, or with 1μM Concanavalin A (ConA) (cat# J61221.MC; Thermo Fisher Scientific) for 5 days. Cultured hamster cells were stained with anti-mouse CD4-APC/Fire750 (clone GK1.5; BioLegend) and anti-mouse CD8-PE (clone 341; Invitrogen) antibodies simultaneously with eBioscience Fixable Viability Dye-efluor-506. Cells were washed and then fixed with 1% paraformaldehyde and ran on the Thermo Fisher Attune NxT, and analyzed with FlowJo Software, V10.

**Data Analysis**

All data were graphed and analyzed by the indicated test using GraphPad Prism Software (San Diego, CA). (*p<0.05, **p<0.01, ***p<0.001, ****p<0.0001)


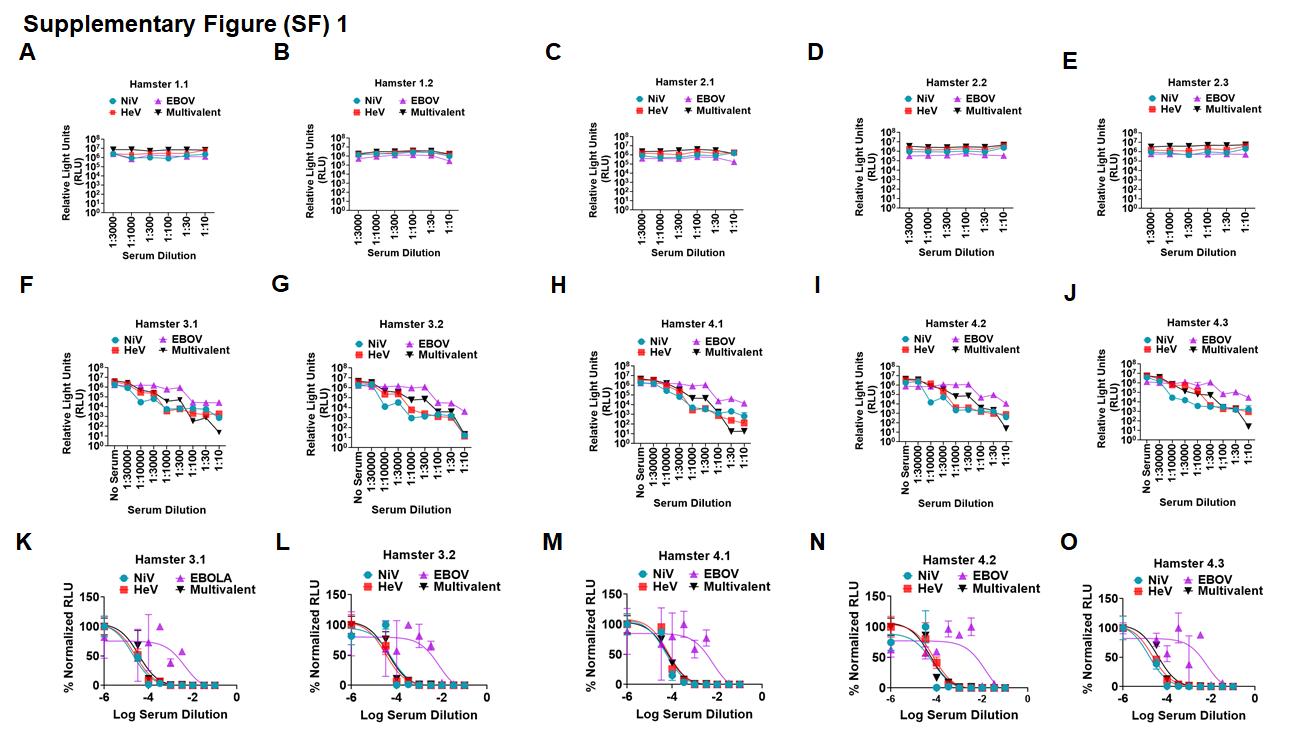


**Supplementary Figure 1: VLPs incorporating NiV F/G, HeV F/G and EBOV GP (multivalent) elicited neutralizing antibodies in hamsters**. **A**. **to E**. Negative control hamsters were vaccinated with bald VLPs and boosted on day 21 and day 42. Serum for the hamsters’ terminal bleed was used to neutralize monovalent NiV F/G, HeV F/G, EBOV GP and the multivalent pseudotyped VSV particles. Entry of VSV particles after neutralization was determined using Renilla Luciferase assay. **F to J**. Monovalent and multivalent pseudotyped VSV were neutralized with different dilutions of sera from hamsters vaccinated with multivalent VLP vaccine. The test vaccine hamster group was vaccinated with the multivalent VLPs on day 0. Vaccination, boosting, serum collection and virus neutralization was done in a similar manner as the negative controls. **K to O**. Normalized graphs for sera from hamsters vaccinated with the multivalent VLPs.

**
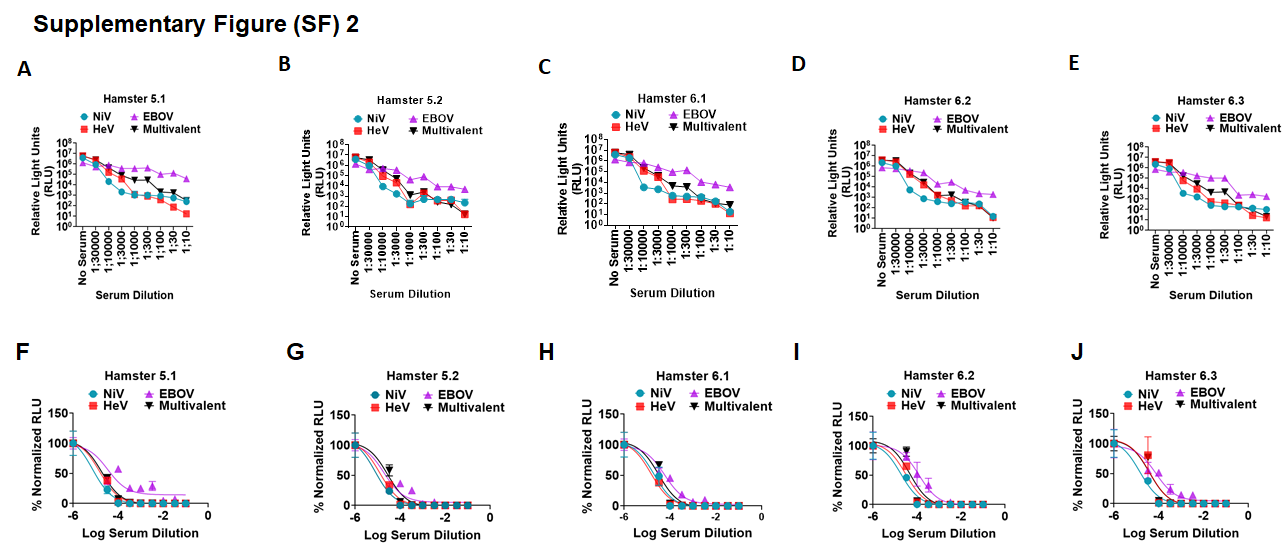
**

**Supplementary Figure 2: Pseudotyped VSV incorporating NiV F/G, HeV F/G and EBOV GP (multivalent) elicited neutralizing antibodies in hamsters. A to E.** Hamsters were vaccinated with the multivalent pseudotyped VSV particles on day 0 and boosted on day 21 and day 42. Serum for the hamsters’ terminal bleed was used to neutralize monovalent NiV F/G, HeV F/G, EBOV GP and the multivalent pseudotyped VSV particles. Entry of the virus into Vero cells following neutralization was analyzed by Renilla Luciferase assay. **F to J.** Normalized graphs for sera from hamsters vaccinated with the multivalent pseudotyped VSV particles.

**
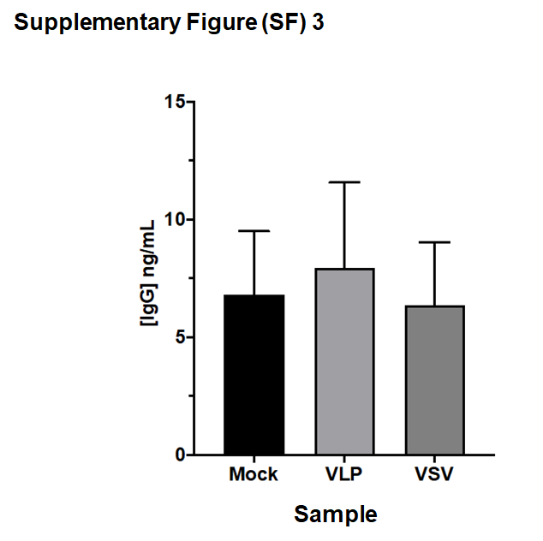
**

**Supplementary Figures 3: Serum IgG ELISA binding quantification between groups that received mock, VLP, or VSV vaccinations.** Hamster serum was diluted 1:10,000 and incubated for 30 minutes on plates provided by Abcam Hamster IgG ELISA Kit (ab200010). Wells were washed 3x using provided buffers and incubated with anti-hamster secondary antibodies for 30 minutes. Development solution was added for 10 minutes, then stopped using the provided stop solution. Quantification was done using a Tecan Spark recording the OD at 450nm.

**
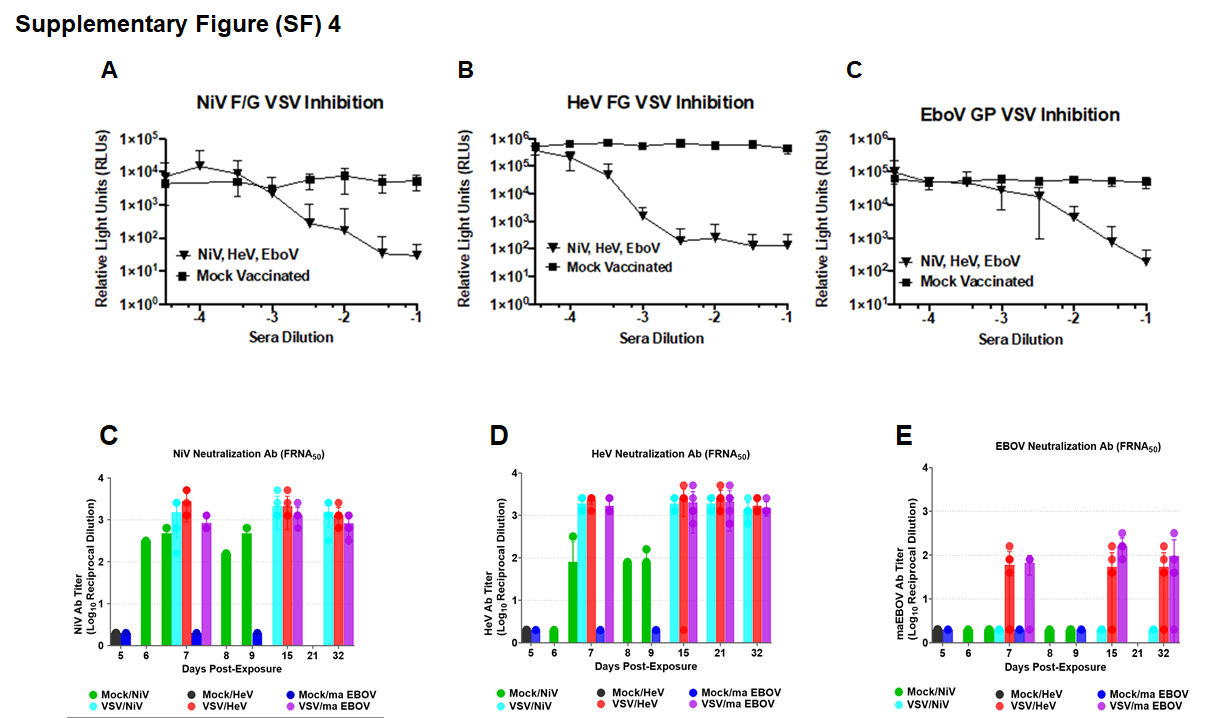
**

**Supplementary figure 4. Determination of neutralizing antibodies against NiV/ HeV and EBOV measured pre and post challenge. A to C.** Neutralization curves for pseudotyped VSV vaccinated hamsters prior to challenge. **A-C** represent neutralizing antibodies measured using NiV, HeV, and EboV VSV virions using sera collected 1 week before challenge ~5 weeks post last vaccination. **D to F**. FRNA50 detecting neutralizing Ab against live NiV/ HeV and EBOV measured pre and post challenge. Average of all animals in each group error bars represent ±SD n=3.

**Supplementary Figure (SF) 5**


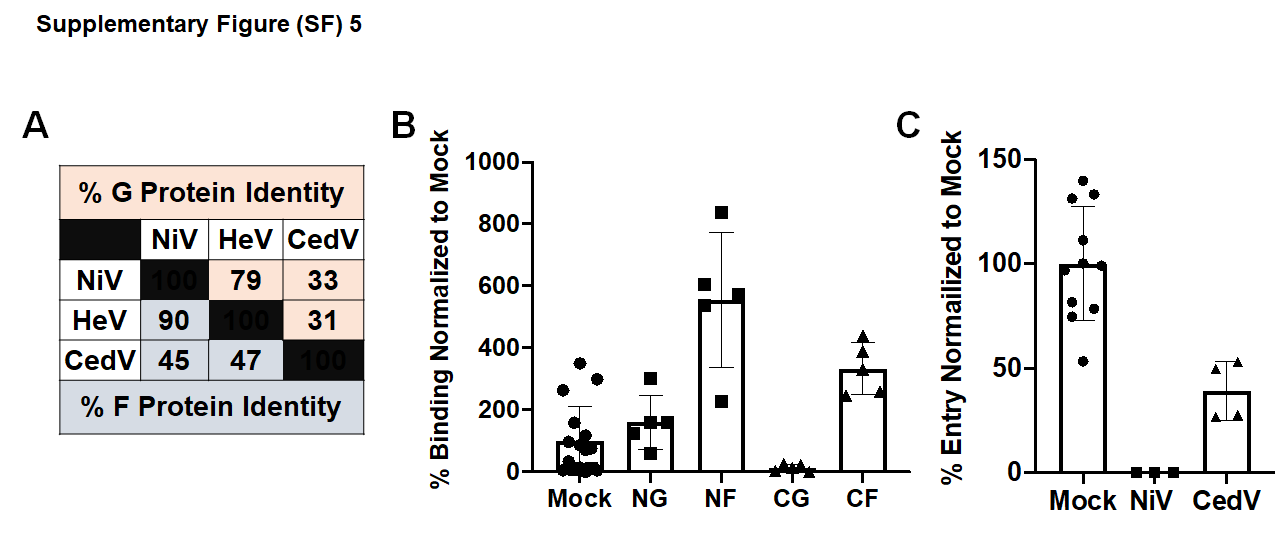


**Supplementary figure 5. Sera from hamsters vaccinated with multivalent pseudotyped VSV virions incorporating NiV F/G, HeV F/G and EBOV GP can bind CedV F and cross neutralize CedV F/G pseudotyped VSV virions**. **A.** Diagram showing sequence identity among NiV, HeV and CedV G proteins. **B.** A comparison of the binding properties of serum to NiV F/G or CedV F/G. **C.** Comparison of entry properties of NiV and CedV pseudotyped VSV virions neutralized with serum from vaccinated hamster 6.2.


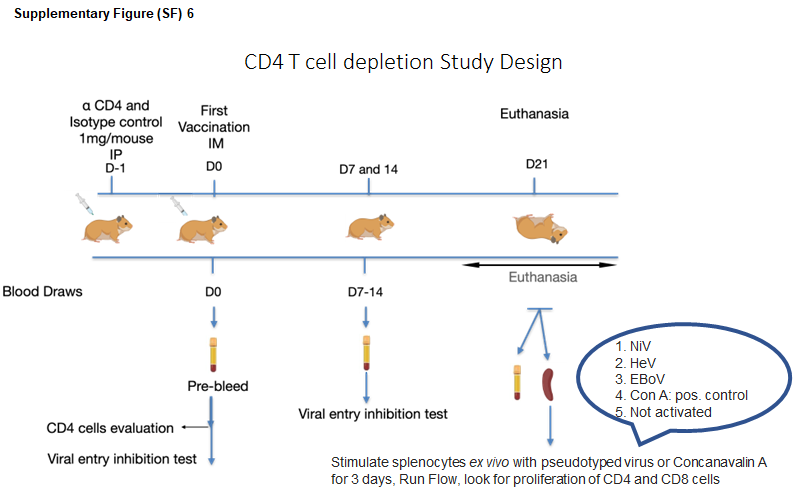


**Supplementary Figure (SF) 6**

**Supplementary Figure 6. CD4^+^ T cell depletion study design.** The figure indicates the period post-vaccination when the hamsters were depleted of the CD4^+^ T cells, vaccinated, bled, and euthanized.

**Supplementary Figure (SF) 7**

**Supplementary Figure 7: Hamster CD4^+^ T cell depletion resulted in sustained decrease in circulating CD4^+^ T cells for 3 weeks.** Hamsters were intraperitoneally injected with 1mg of either isotype control or anti-CD4^+^ antibodies one day prior to vaccination. Blood was collected 24 hours post depletion (Day 0), and each subsequent week through their euthanasia on Day 20. CD4^+^ T cells were graphed as percent of live cells, and then normalized to hamsters that did not receive antibodies, mock-injected at Day -1. (n=3); Two-Way ANOVA with Sidak’s multiple comparison test (*p<0.05, **p<0.01, ***p<0.001, ****p<0.0001).


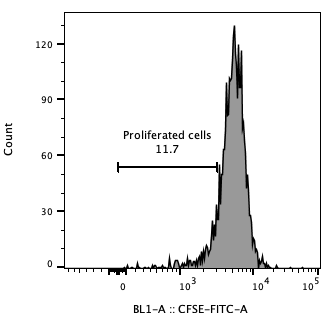

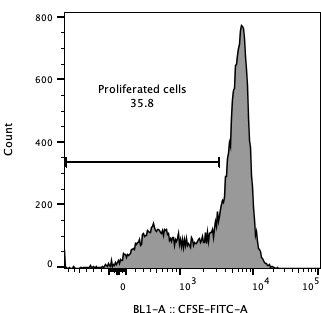

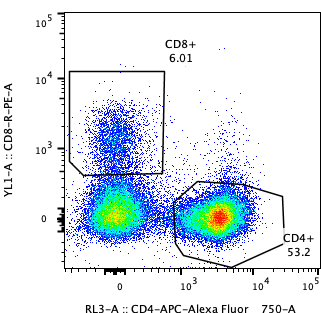

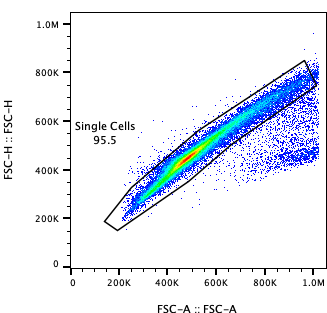

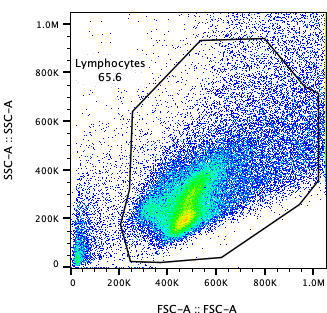

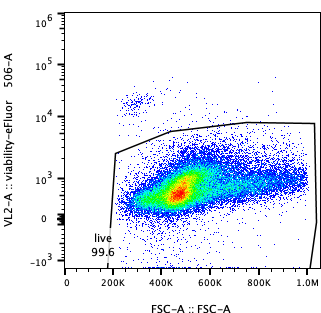


**Supplementary Figure (SF) 8**

**Supplementary Figure 8:** Identification of proliferated CD4^+^ and CD8^+^ T cells. Gating strategy of proliferated CD8^+^ and CD4^+^ T cells from *ex* vivo splenocyte culture. Lymphocytes were gated from the forward and side scatter plot (A), and then doublets were excluded on the forward scatter area vs height plot (B). Next, live cells were gated (C) and then split into CD4^+^ or CD8^+^ cells (E), and finally, CD8^+^ and CD4^+^ cells were measured for proliferation (D & F, respectively) through the decrease of CFSE staining.

**Supplementary Figure (SF) 9**

A

B

**Supplementary Figure 9: CD4^+^ T cell depletion during vaccination prevents CD4^+^ and CD8^+^ cell restimulation to vaccine antigens.** CD4^+^ T cells (grey), or Control (black) were depleted from hamsters 24 hours once prior to vaccination with multivalent VSV pseudotyped virus. Animals were euthanized 20 days post vaccination, and total splenocytes were stained with CFSE and cultured for 5 days in the presence of media alone (UnAct), pseudotyped bald virus control (PBC), monovalent NiV F/G (NiV), HeV F/G (HeV), EBoV GP (EBoV), or multivalent pseudotyped viruses, or with 1μM ConA stimulation as a positive control. Proliferation of CD4^+^ (A) or CD8^+^ (B) T cells were analyzed via flow cytometry, and percent of proliferated cells were graphed and analyzed with Two-Way ANOVA and Sidak’s multiple comparison test (*p<0.05, **p<0.01, ***p<0.001, ****p<0.0001) (n=3).

**Supplementary Figure (SF) 10**

**Supplementary Figure 10: CD4+ T cell depletion during vaccination does not limit neutralizing antibody titers.** CD4^+^ cells were depleted from hamsters 24 hours prior to vaccination with multivalent VSV pseudotyped particle vaccine. Weekly serum from non-vaccinated, isotype control treated, or CD4^+^ T cell-depleted hamsters was used to neutralize monovalent NiV F/G (left column), HeV F/G (middle column), EBoV GP (right column) VSV particles and entry of the virus into cells was analyzed by Renilla Luciferase assay. The mean neutralization activity for each pseudotyped particle vaccine was calculated and normalized to day 0 (pre-vaccinated) serum. Finally, curves were generated in Prism as non-linear inhibitor vs normalized . Errresponse data. Error bars show standard deviation.


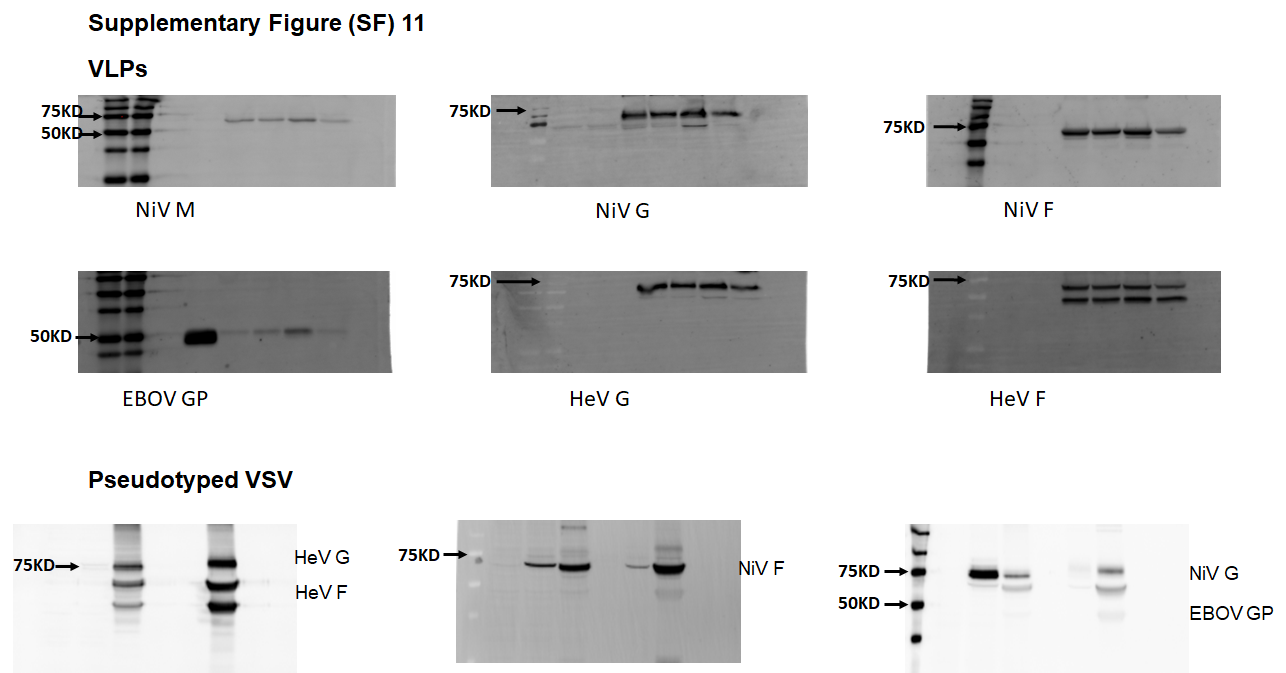


**Supplementary Figure 11: Optimized production of NiV-HeV-EBOV VLPs and Pseudotyped VSV analyzed by Western blotting.**
